# proBAMsuite, a Bioinformatics Framework for Genome-Based Representation and Analysis of Proteomics Data*[Fn FN2]

**DOI:** 10.1074/mcp.M115.052860

**Published:** 2015-12-11

**Authors:** Xiaojing Wang, Robbert J. C. Slebos, Matthew C. Chambers, David L. Tabb, Daniel C. Liebler, Bing Zhang

**Affiliations:** From the ‡Department of Biomedical Informatics,; §Department of Biochemistry,; ‖Department of Cancer Biology, Vanderbilt University School of Medicine, Nashville, TN 37232;; ¶Jim Ayers Institute for Precancer Detection and Diagnosis, Vanderbilt-Ingram Cancer Center, Nashville, TN 37232

## Abstract

To facilitate genome-based representation and analysis of proteomics data, we developed a new bioinformatics framework, *proBAMsuite*, in which a central component is the protein BAM (*proBAM*) file format for organizing peptide spectrum matches (PSMs)^1^ within the context of the genome. *proBAMsuite* also includes two R packages, *proBAMr* and *proBAMtools*, for generating and analyzing *proBAM* files, respectively. Applying *proBAMsuite* to three recently published proteomics datasets, we demonstrated its utility in facilitating efficient genome-based sharing, interpretation, and integration of proteomics data. First, the interpretation of proteomics data is significantly enhanced with the rich genomic annotation information. Second, PSMs can be easily reannotated using user-specified gene annotation schemes and assembled into both protein and gene identifications. Third, using the genome as a common reference, *proBAMsuite* facilitates seamless proteomics and proteogenomics data integration. Finally, *proBAM* files can be readily visualized in genome browsers and thus bring proteomics data analysis to a general audience beyond the proteomics community. Results from this study establish *proBAMsuite* as a useful bioinformatics framework for proteomics and proteogenomics research.

Mass-spectrometry-based shotgun proteomics technology has undergone rapid advancements during the past decade. Recent studies have demonstrated deep proteome coverage with the identification of more than 10,000 proteins (1–5). Moreover, large-scale integrative proteogenomic studies have started to harness the complementary strengths of the proteomics and genomics technologies (6–8). To facilitate the exchange and sharing of the rapidly growing body of proteomics data, the Human Proteome Organization Proteomics Standards Initiative has defined community standards for data representation, including standard data formats for reporting peptide and protein identification results (9). However, although peptide and protein identification relies primarily on protein databases derived from the reference genome sequence, genomic locations of identified peptides are not reported by commonly used mass spectrometry data analysis software, which limits genome-based interpretation and analysis of proteomics data and hinders effective proteogenomic data integration.

First, without knowing genomic locations of the identified peptides, some important questions are left hanging. For example, peptides that map to multiple proteins introduce ambiguity in protein inference. Those mapping to the same genomic locus can benefit from a gene-level instead of a protein-level inference; however, it is unclear how many and which peptides map to multiple proteins derived from the same genomic locus. As another example, exon–exon junction peptides are important for the understanding of alternative splicing and protein isoform complexity, but it is difficult to determine how many and which peptides span more than one exon with existing data formats. Furthermore, although a major goal in proteomics is to achieve a comprehensive coverage of the coding genome, calculating the sequence coverage ratio for the whole coding genome is cumbersome with existing data formats.

Second, with proteins serving as the data organization unit in a data analysis report, it is difficult to perform data integration across multiple proteomics studies. Studies may use different reference protein databases with inconsistent protein annotations for database searching, thus data integration usually requires re-searching of the raw data against a common reference database. In addition, although gene-centric reports are required by many downstream pathway and network analysis tools, additional efforts are required to derive them from protein-centric reports.

Moreover, it remains difficult to communicate proteomics data to the genomics community. Integrating a protein-centric report with data generated from genomics or transcriptomics analyses is a barrier to proteogenomic analysis. As proteogenomics is rapidly becoming an attractive and important research field (10–13), it is critical to have a new data format and supporting tools that enable smooth integration across proteomics, genomics, and transcriptomics data.

Recently, several software tools have been published to facilitate the visualization of peptides in genome browsers, including iPiG (14), CAPER (15), and PG Nexus (16), among others (17–19). These tools address a critical need of genome browser-based visualization of proteomics data; however, although a genome-based representation of proteomics data introduces novel data analysis and interpretation opportunities that go beyond visualization; these opportunities have barely been explored. In a recent study, the sequence alignment/map (SAM) file format developed in the next-generation sequencing field was adopted in the tool PG Nexus (16) to allow covisualizing proteomic data with genomes and transcriptomes. Nevertheless, although a primary goal of the SAM format is to provide a well-defined interface between sequence alignment and downstream analyses (20), this important feature has not been exploited in PG Nexus. Moreover, there has been no attempt to incorporate proteomics-specific information into the SAM format.

To provide an integrated solution to facilitate genome-based representation and analysis of proteomics data, we developed *proBAMsuite*. A central component of *proBAMsuite* is the protein BAM (*proBAM*) file format for storing and analyzing peptide spectrum matches (PSMs) within the context of the genome. *proBAM* is built upon the success of the SAM format and its compressed binary version, BAM (20), with necessary modifications to accommodate information specific to proteomics data such as PSM scores, charge states, and protein modifications. *proBAMsuite* also includes two R packages, *proBAMr* and *proBAMtools*, for generating and analyzing *proBAM* files, respectively. Applying *proBAMsuite* to three recently published proteomics datasets, we demonstrate its utility in facilitating efficient genome-based sharing, interpretation, and integration of proteomics data.

## MATERIALS AND METHODS

### 

#### 

##### Definition of the proBAM File Format

The *proBAM* format is adapted from the BAM format and contains a header section and an alignment section. A full description of the BAM format is available at http://samtools.github.io/hts-specs/SAMv1.pdf. The fundamental difference between *proBAM* and BAM is that PSMs replace sequence reads as the basic data unit in the *proBAM* format. Moreover, novel mandatory fields are introduced in the alignment section to accommodate unique features of proteomics data. For instance, we introduce the “XM” field to store peptide modification information, the “XS” field to store PSM scores, and the “XC” field to store peptide charge state information. All fields follow the “TAG:TYPE:VALUE” format, which is similar to those in a BAM file. In the future, additional fields can be easily added to accommodate specific needs from the proteomics community. *proBAM* allows for five bitwise flag values to describe peptide mapping information. A detailed description of the *proBAM* format is available in Supplemental Table S1, which includes the list of all mandatory fields, the definition and value format for each of the new fields introduced in *proBAM*, and the five FLAG values.

##### Generation of proBAM Files

The pipeline for generating *proBAM* files from proteomics data is illustrated in Supplemental Fig. S1. We developed the R package *pepXMLTab* (http://www.bioconductor.org/packages/release/bioc/html/pepXMLTab.html) to convert the “spectrum query” section of a pepXML file to a data frame and to filter the PSMs based on a user-specified PSM false discovery rate (FDR) threshold. Other proteomics data formats (*e.g.* mzIdentML) can be converted to pepXML first using tools such as the ProteoWizard (21). We developed the R package *proBAMr* (http://www.bioconductor.org/packages/release/bioc/html/proBAMr.html) to map peptides back to the genome (Supplementary File 1). *proBAMr* generates SAM files, which are subsequently converted to the binary BAM format and indexed for fast access using SAMtools (20).

**Fig. 1.**
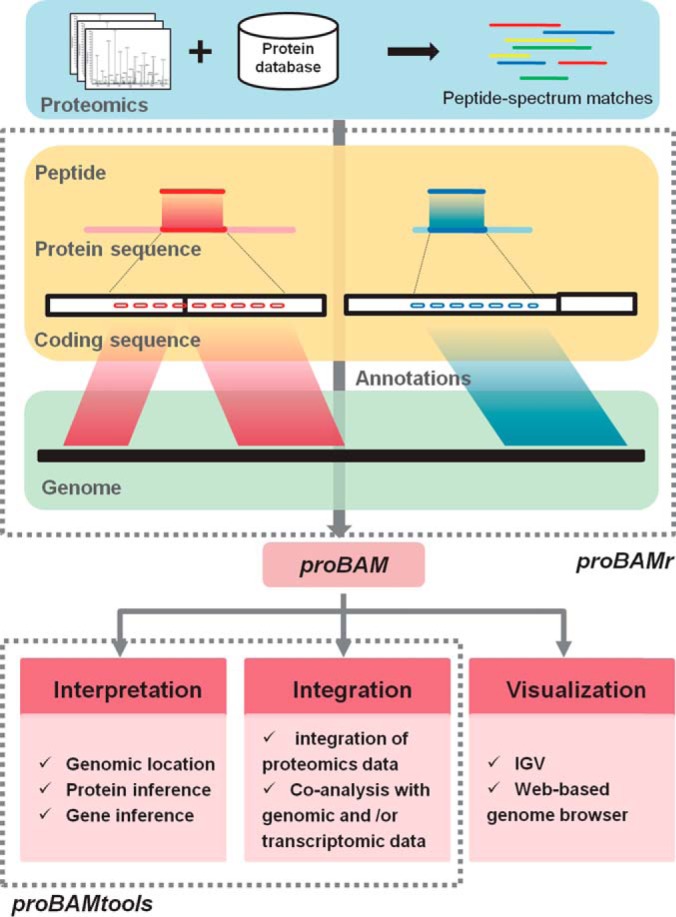
**Overview of the *proBAMsuite*.** Peptide-spectrum matches (PSMs) resulting from proteomics database search are mapped to the genome and the mapping information is stored in the *proBAM* format, which facilitates genome-based interpretation, integration, and visualization of proteomics data. Two R packages, *proBAMr* and *proBAMtools*, were developed for generating and analyzing the *proBAM* files, respectively. The mapping procedure of *proBAMr* is described in the Methods section. *proBAMtools* includes functions for genome-based proteomics data interpretation and integration (see Supplemental Fig. S1 for details).

The mapping procedure of *proBAMr* consists of three steps (Fig. 1). First, the peptide from a PSM is mapped to corresponding protein sequence, and its starting and ending positions in the protein sequence are recorded. Second, the coding sequence of the peptide is retrieved from the protein coding sequence according to the starting and ending positions. Finally, genome coordinates for the PSM are determined based on the gene structure and genomic location of the protein coding gene. Peptides mapped to reverse sequences in the “decoy” database are also kept in the *proBAM* file to facilitate the estimation of protein and gene-level FDRs. These peptides are mapped to the genomic locations of their corresponding forward sequences in the reverse direction. A very small number of peptides may not be correctly linked to a genomic location using this procedure, usually because the presumed encoding sequence is not consistent with the peptide sequence. This likely reflects incorrect annotation of related regions.

Running time of the mapping procedure depends largely on the number of identified PSMs in a pepXML file and the protein sequence database used. For the Clinical Proteomic Tumor Analysis Consortium colorectal cancer dataset (CPTAC_CRC), the Technische Universitat Munchen_ National Cancer Institute 60 cell line dataset (TUM_NCI60), and the Vanderbilt University Colorectal Cancer 10 cell line dataset (VU_CRC10), the median runtime for a single pepXML file was 43, 91, and 140 min, and the Random-access memory (RAM) usage was 0.4, 0.4, and 0.7 GB, respectively. The mapping of PSMs from different pepXML files can be easily parallelized.

##### proBAMtools

We developed an R package, *proBAMtools* (http://bioinfo.vanderbilt.edu/proteogenomics/), to perform various analyses based on the *proBAM* files. *proBAMtools* includes functions for genome-based proteomics data interpretation, protein and gene inference, count-based quantification, and data integration (Fig. 1, Supplemental Fig. S1, Supplementary File 1).

##### Protein and Gene Inference

**Fig. 2.**
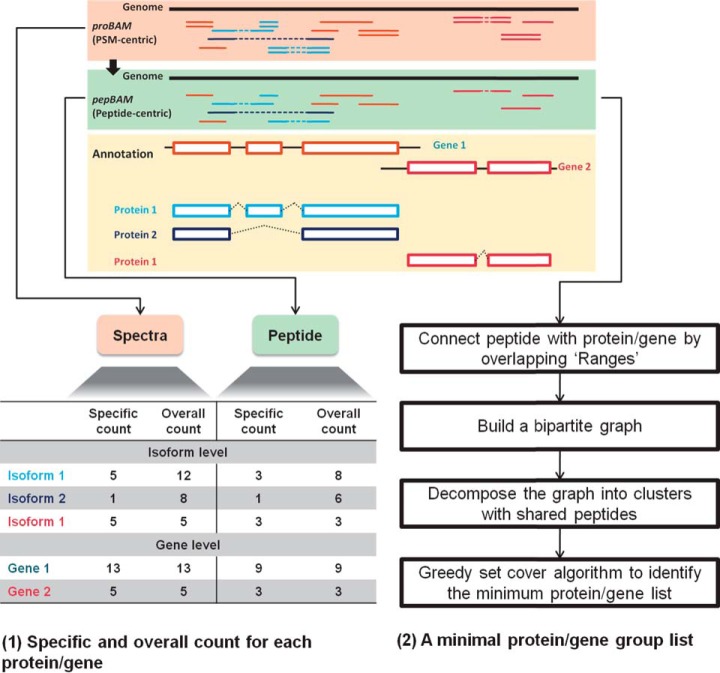
**Schematic overview of *proBAM*-based protein and gene inference and peptide and spectra counting.** A peptide-centric representation of proteomics data (green background box) is generated from *proBAM* (orange background box) using functions provided in *proBAMtools*. A hypothetical example for two genes is presented in the workflow (yellow background box). Gene 1 has two protein isoforms whereas Gene 2 has only one protein isoform. Peptide spectrum matches (PSMs) and peptides associated with Gene 1 are visualized in three different colors based on whether they are shared by both protein 1 and 2 (orange) or specific to one protein (light blue or dark blue). The table (lower left) summarizes the spectral and peptide counts for each protein or gene, including both protein- or gene-specific counts and overall counts. Meanwhile, the parsimony procedure, demonstrated at lower right, is applied to generate the minimum protein and gene group lists. The major improvement is that *proBAMtools* assigns peptides by overlapping the mapped peptides with gene structures, thus enabling both protein and gene level inference.

*proBAMtools* uses a previously published parsimony procedure (22) to generate a minimal list of identified proteins or genes (Fig. 2). The procedure relies on bipartite graph modeling. What distinguishes the *proBAM* approach from the previous approach is the utilization of genomic location information in the construction of the bipartite graph. Specifically, using the Range infrastructure developed by the genomics community (23), the *proBAM*-based approach can connect peptides to different types of genomic elements such as exons, transcript isoforms, or genes based on their overlapping relationship on the genome. Consequently, inference can be made at both the protein level and the gene level. Moreover, inference can be made based on different versions of genome annotations. After the parsimony analysis, a minimum of two peptides is required to identify a protein or gene group. Because the PSMs of decoy peptides are kept in *proBAM* files and are included in protein and gene inference, protein- and gene-level FDRs can be calculated using the target-decoy strategy.

##### Count-Based Quantification

*proBAMtools* generates count tables on the basis of genomic structure of the genes. Both spectral count and peptide count tables are provided at protein isoform and gene levels, respectively, and both overall counts and gene-specific or isoform-specific counts are reported (Fig. 2). The overall spectral count for a gene or an isoform, respectively, sums up all PSMs associated with the gene or the isoform, whereas the gene-specific or isoform-specific spectral count, respectively, sums up only PSMs associated specifically with the gene or the isoform. The same conditions also apply to peptide counting. Count data are provided for individual genes and proteins. By integrating these data with the minimal protein or gene group lists resulting from the parsimony analysis, count tables can be generated for the confidently identified genes or proteins.

##### Reannotation of PSMs

*proBAMtools* uses a three-step procedure to reannotate PSMs according to a user-specified gene annotation scheme by filtering out PSMs that are not supported by the gene annotation scheme (Supplemental Fig. S2*A*). First, PSMs mapping to regions out of the coding DNA sequences (CDSs) of the user-specified annotation scheme are removed. Second, PSMs mapping to regions inconsistent with the gene structures of the user-specified scheme are removed. Finally, PSMs with peptides out of frame in the user-specified scheme are removed. All remaining PSMs can be associated with the user-specified gene annotation scheme.

##### Proteomics Data Integration

**Fig. 3.**
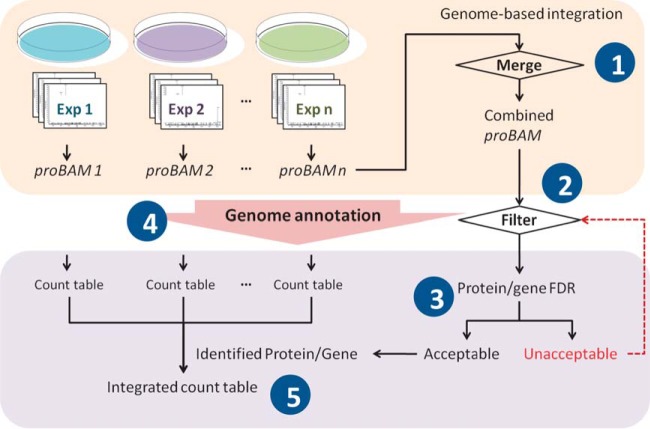
**proBAM based proteomics data integration procedure.** In *proBAM* files, PSMs are aligned to the genome regardless of the protein database used in each proteomics study. The integration procedure therefore involves: 1) merging the *proBAM* files from individual studies; 2) choosing a genome annotation scheme and a PSM FDR for protein and gene inference; 3) calculating protein and gene FDRs (repeating step 2 with a more stringent PSM FDR enables refinement of gene and protein FDRs); 4) generating count tables for individual studies; and 5) generating an integrated count table for all confidently identified protein and gene groups.

Figure 3 depicts the data integration procedure implemented in *proBAMsuite. proBAM* files are generated from individual studies using *proBAMr* based on user-specified PSM FDRs. Because PSMs in all resulting *proBAM* files are mapped to the genome and thus aligned in the same coordinate system, they easily can be combined into one *proBAM* file. All PSMs in this combined *proBAM* file can be reannotated according to a user-specified gene annotation scheme as described above. Protein and gene inference as well as count tables can be generated using *proBAMtools*. Notably, protein- and gene-level FDRs may increase dramatically with increased sample size. Therefore, if the protein- and gene-level FDRs are unacceptable, reanalysis using a strengthened PSM FDR or other additional filters (*e.g.* count per protein or gene) should be performed.

##### Proteomics Datasets and Peptide Identification

Three recently published proteomics datasets were used in this study, including the CPTAC_CRC dataset (8), the TUM_NCI60 dataset (4), and the VU_CRC10 dataset (24).

For the CPTAC_CRC dataset (8), we downloaded the idpDB file from the CPTAC data portal (https://cptac-data-portal.georgetown.edu/cptac/s/S022). This file was generated from a custom assembly based on RNA-Seq derived protein databases. For each sample, we generated one *proBAM* file, which included both normal and variant peptides. For visualization in the proteogenomics browser (http://bioinfo.vanderbilt.edu/proteogenomics), two aggregated *proBAM* files were generated. One includes all normal and variant peptides identified from all samples, whereas the other includes only variant peptides from all samples.

For the TUM_NCI60 dataset (4), we downloaded raw files from ftp://129.187.44.58/share/nci60/raw/ProteomeProfiles/. Only data from “Proteome Profiles” was used in this study, resulting in 61 datasets from 59 cell lines. The downloaded .mz5 files were converted into mzML files. We used the GENCODE v19 human protein database (ftp://ftp.sanger.ac.uk/pub/gencode/Gencode_human/release_19/) to identify peptides. MyriMatch version 2.1.138 (25) was used for database search. MyriMatch employed precursor tolerances of 10 ppm and allowed fragments to vary by up to 0.6 *m/z*. Semi-tryptic peptides were considered equally with fully tryptic peptides, and matches allowed for isotopic error in precursor ion selection. Searches conducted on-the-fly peptide sequence reversal and applied static +57 modifications to cysteines and dynamic +16 oxidations to methionines. MyriMatch also added pyroglutamine modifications to the N termini of peptides starting with Gln residues. A minimum peptide length of 6 was required. After database searching, we used the R package *pepXMLTab* to generate tabular files containing the PSMs with FDR less than 0.01 in individual pepXML files. *proBAM* files were generated for individual cell lines and then aggregated by tissue origin to create nine *proBAM* files for visualization in the proteogenomics browser. For the demonstration of *proBAM*-based switching of gene annotation scheme, the dataset was also searched against the RefSeq human protein sequence database (download from The University of California Santa Cruz (UCSC) table browser as of October 24, 2013) using MyriMatch as described above.

For the VU_CRC10 dataset, previously published proteomics data from 10 colorectal cancer cell lines (24) were searched against a customized protein sequence database generated from paired RNA-Seq data using *customProDB* (26). MyriMatch version 2.1.138 was used for database search and was configured to consider both fully tryptic and semi-tryptic peptide matches with a precursor mass/charge (*m/z*) tolerance of 1.5 and a fragment *m/z* tolerance of 0.5. Carboxamidomethylation of cysteines was included as static modification while methionine oxidation and N-terminal pyroglutamines were included as a dynamic modification in the searches. A minimum peptide length of 6 was required. Then *pepXMLTab* was used to generate tabular files that contain the PSMs with FDR less than 0.01 in each pepXML file. By including variations and novel junctions in the customized databases, we were able to identify variant peptides and novel junction peptides from the proteomics datasets. For each cell line, a *proBAM* file was generated, which includes normal peptides, variant peptides, and novel junction peptides.

## RESULTS

### 

#### 

##### Data Sharing and Visualization

The *proBAM* file format facilitates public sharing and re-use of mass-spectrometry-based proteomics data. We demonstrate its utility using three proteomics datasets: 1) CPTAC_CRC: proteomics data for 91 samples representing 86 The Cancer Genome Atlas colorectal cancer (CRC) tumors generated by the Vanderbilt Proteome Characterization Center in the National Cancer Institute Clinical Proteomic Tumor Analysis Consortium (CPTAC) (8); 2) TUM_NCI_60: proteomics data for 61 samples representing 59 NCI-60 cell lines generated at the Technical University of Munich (4); and 3) VU_CRC_10: proteomics data for 10 CRC cell lines generated at the Vanderbilt University School of Medicine (24). We converted PSMs from these studies to the *proBAM* format using the *proBAMr* package. The compact size of *proBAM* files facilitates easy data exchange. For example, the aggregated *proBAM* file of the CPTAC_CRC dataset is 64 MB, which is more than 100 times smaller in size compared with the original idpDB report (8.7 GB) from IDPicker3 (27). All three datasets have been preloaded in a JBrowse (28)-based genome browser (http://bioinfo.vanderbilt.edu/proteogenomics), which offers easy access of these data to a broad audience both within and outside the proteomics community. As shown in Supplemental Fig. S3, peptide evidence for alternative transcript isoforms, exon–exon junctions, and mutations can be easily retrieved and visualized.

##### Genome-Based Proteomics Data Interpretation

**Fig. 4.**
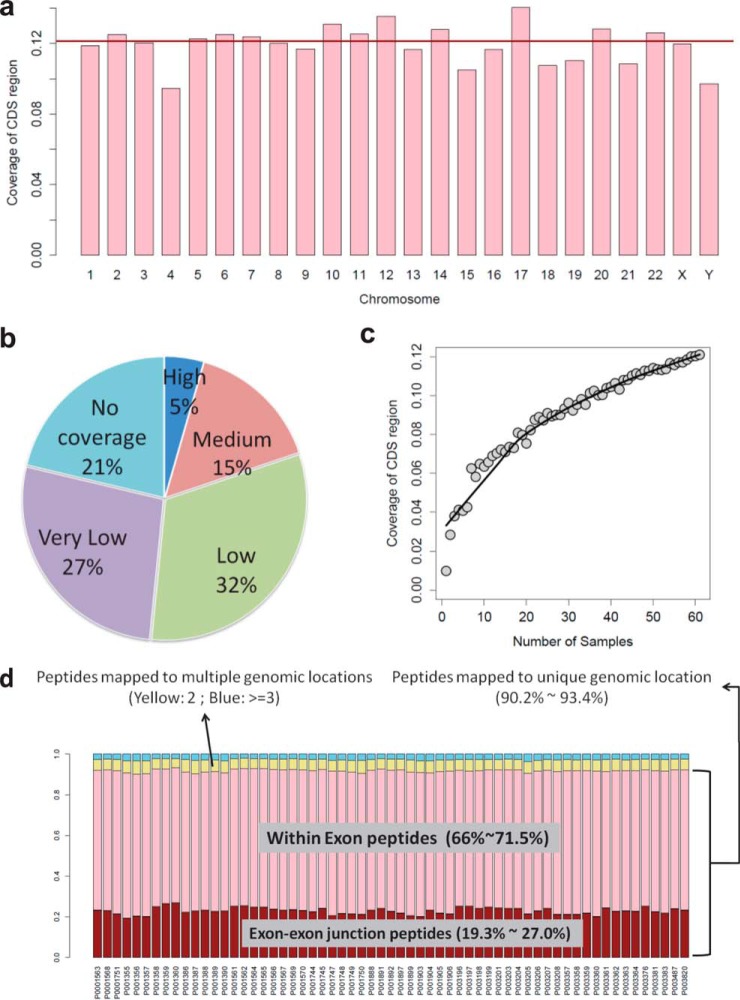
**Proteome coverage and the genomic location of identified peptides of the TUM_NCI_60 dataset.** (*A*) Coding DNA sequence (CDS) coverage for individual chromosomes. The red line represents the average genome CDS coverage. (*B*) The distribution of 20,738 genes across different CDS coverage categories. Genes are divided into five categories based on the level of CDS coverage: no coverage, very low coverage (0–5%), low coverage (5–20%), medium coverage (20–50%), and high coverage (>50%). (*C*) Cumulative distribution of whole genome CDS coverage as a function of sample size. Each dot represents a random subset of samples. The black line represents a smoothed fit. (*D*) Classification of identified peptides according to genomic location information. Peptides are separated into those mapped to multiple genomic locations (blue and yellow) and a unique genomic location (pink and red). The latter group is further separated into exon–exon junction peptides (red) and within exon peptides (pink).

One unique feature of the *proBAMsuite* is the enabling of genome-based interpretation of proteomics data. Whereas existing data formats only report sequence coverage for individual proteins, the *proBAM* format stores the genomic locations of peptides and thereby allows *proBAMtools* to calculate sequence coverage at the genome, chromosome, and individual gene levels. Using the aggregated TUM_NCI_60 dataset as an example, *proBAMtools* reported coverage of 12% for the whole human coding genome. All chromosomes were evenly covered in their coding regions in general, although chromosomes 4 and Y had relatively lower coverage (*p* = 0.027 and 0.046, respectively, z-test, Fig. 4*A* and Supplemental Fig. S4). The low Y chromosome coverage might be due to the fact that 24 out of the 59 cell lines are female origin. Proteomics evidence was found for 79% of the protein-coding genes, but only 5% of the genes had sequence coverage over more than 50% of their coding regions. The percentages of genes with 0–5%, 5–20%, and 20–50% of sequence coverage were 27%, 32%, and 15%, respectively (Fig. 4*B*). We next investigated how sample size affects sequence coverage for the whole coding genome. Using a resampling strategy (Fig. 4*C*, Supplementary Note, and Supplemental Fig. S5), we observed a clear benefit of adding more samples to increase the coding genome coverage, although the increase became relatively smaller when the sample sizes were larger than 20 (Fig. 4*C*). Combining the VU_CRC_10 dataset increased the coding genome coverage from 12% to 15%, and further combining the CPTAC_CRC dataset increased the coverage to 18%. Such information and the ability to perform these analyses are particularly useful in projects aiming at a complete genome-wide proteome characterization (29, 30).

In a typical proteomics study, many identified peptides can be attributed to more than one protein. These multiple-mapping or shared peptides introduce ambiguity and complicate protein inference (22, 31). Shared peptides likely originate from two different sources-different transcript isoforms encoded by the same gene or different genes with high sequence similarity, such as homologous genes in a conserved gene family or genes with a common conserved protein domain. In the first scenario, shared peptides would map to the same genomic location, whereas in the second scenario, shared peptides map to different loci. *proBAMtools* can classify all identified peptides into different categories based on whether they map to one or multiple positions in the genome. Across all cell lines in the TUM_NCI_60 dataset, we found that 90.2–93.4% of the distinct peptide sequences were mapped to a unique genomic location, 4.5–6.6% mapped to two locations, 1.2–1.8% mapped to three locations, and 2.1–3.8% mapped to three or more locations (Fig. 4*D* and Supplemental Table S2). This result suggests that shared peptides resulted primarily from different transcript isoforms encoded by the same genes, which prompted us to implement gene-level inference of proteomics data in *proBAMtools* to reduce ambiguity (see next section).

Exon–exon junction peptides can provide direct evidence for alternative splicing but cannot be recognized easily with existing data formats. *proBAMtools* can distinguish within exon peptides from exon–exon junction peptides. Among the peptides mapped to a unique genomic location, the fraction of junction peptides was 19–27% (25.8% in the merged *proBAM* file) across cell lines in the TUM_NCI_60 dataset (Fig. 4*D*). This partially explains the low peptide identification rates when using solely the six-frame translated human genome database for proteomics search (32). The majority of the exon–exon junction peptides spanned two exons (26,851 out of 27,564, 97.41% in the merged *proBAM* file), whereas a small number spanned more than two exons (Supplemental Table S2).

##### Gene-Level Inference versus Protein-Level Inference

*proBAMtools* supports both protein-level inference and gene-level inference. Using the cell line HCT116 in the TUM_NCI_60 dataset as an example, after the parsimony procedure and single-hit group removal, we identified 740 protein groups and 743 gene groups. Although 712 (96%) of the gene groups contained only one gene, only 272 (37%) protein groups contained one protein. Similar results were obtained in all cell lines, suggesting that most of the proteins cannot be unambiguously identified and that gene-level inference reduces ambiguity.

**Fig. 5.**
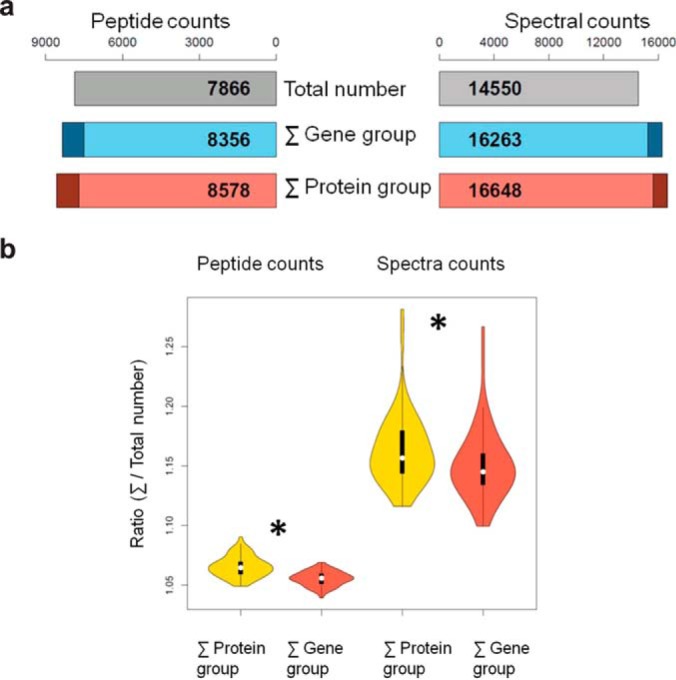
**Evaluation of the overcounting problem in protein-level or gene-level inferences.** (*A*) Comparison of the peptide and spectral count sums for gene groups in gene-level inference and those for protein groups in protein-level inference, using data from cell line HCT116 in the TUM_NCI_60 dataset. Gray bars represent the actual total number of identified peptides (left) or spectra (right), respectively. Dark-colored regions of red and blue bars represent peptides or spectra that were removed because they mapped to single-hit proteins or gene groups. Gene-level inference yielded slightly lower count sums when compared with protein-level inference. (*B*) Similar to *A*, each violin plot summarizes data from all cell lines in the TUM_NCI_60 dataset. Gene-level inference yielded lower aggregated peptide count and spectra count compared with protein-level inference (*p* < 0.01, paired *t* test).

When reporting identified proteins and their supporting peptides, a minimal list of proteins, including both distinct and differentiable proteins, are usually provided to explain all observed peptides (31). A single spectrum associated with multiple protein groups could complicate the spectral-counting-based quantification procedure. Some simple algorithms count these spectra multiple times (22), whereas more advanced algorithms adjust spectral counts of shared peptides by leveraging information from unique peptides (33). Although promising, apportioning a large number of spectra on the basis of relatively small sets of differentiating spectra remains a challenge and may lead to overfitting. Using gene-level assembly provides an alternative to alleviate the shared peptide problem. For the cell line HCT116, we identified a total of 7,866 distinct peptides and 14,550 spectra (Fig. 5*A*). The peptide counts for individual protein groups summed to 7,707. Adding the 871 peptides from the removed single-hit protein groups resulted in a total of 8,578 peptides, which was 9% more than the total number of identified peptides. In contrast, the peptide counts of individual gene groups summed to 7,495. Adding the 861 peptides from the removed single-hit gene groups resulted in a total of 8,356 peptides, which was 6% more than the total number of identified peptides. Similarly, protein-level and gene-level inferences overestimated the total number of identified spectra by 14% and 12%, respectively. Analysis using data from all cell lines in the TUM_NCI_60 dataset showed that gene-level inference yielded significantly lower aggregated peptide and spectrum counts compared with protein-level inference (*p* < 0.01, paired *t* test, Fig. 5*B*). These results suggest that gene-level quantification helps reduce the number of shared peptides and thus reduces the complexity in spectral counting.

##### Easy Reannotation of PSMs

**Fig. 6.**
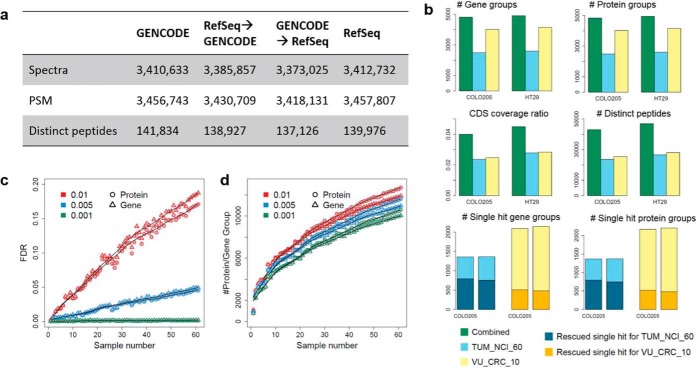
**proBAM enables proteomics data integration.** (*A*) The numbers of identifiable spectra, peptide-spectrum matches (PSMs), and distinct peptides from the TUM_NCI_60 dataset. The three columns represent search results based on: 1) the GENCODE database; 2) the GENCODE database, and then reannotated to RefSeq annotation using *proBAMtools*; and 3) the RefSeq database. Similar results in columns 2 and 3 indicate that *proBAM* can facilitate easy reannotation of PSMs without new database searching. (*B*) Proteomics data integration for cell lines COLO205 and HT29, which were included in both the TUM_NCI_60 dataset searched against the GENCODE protein database and the VU_CRC_10 dataset searched against customized ENSEMBL66 databases. Merged proteomic identifications were analyzed based on the GENCODE annotation using *proBAMtools*. Blue, yellow, and green bars represent results from the TUM_NCI_60 dataset, the VU_CRC_10 dataset, and the combined dataset, respectively. The two top figures show the numbers of identified gene groups and protein groups, respectively. The two middle figures show CDS coverage ratios and the numbers of distinct peptides, respectively. The two bottom figures show the numbers of single-hit gene groups and protein groups, respectively, with the dark-colored sections representing gene and protein groups that acquired additional unique peptides through data integration. The number of rescued gene/protein group is defined as how many groups do not overlap with each dataset after integration. (*C*) Protein and gene FDRs as a function of sample size in the TUM_NCI_60 dataset. Data from different PSM FDRs are plotted in different colors. (*D*) The numbers of identified protein and gene groups as a function of sample size in the TUM_NCI_60 dataset. Data from different PSM FDRs are plotted in different colors.

PSMs in a *proBAM* file are aligned against the genome regardless of the protein database used for peptide identification, allowing easy reannotation of the PSMs using different gene annotation schemes. To illustrate this point, we searched the TUM_NCI_60 dataset against two reference databases, RefSeq and GENCODE, respectively. Using *proBAMtools*, we converted the GENCODE-based search results to RefSeq annotation and *vice versa*. The process retained 98.9% of PSMs converting from GENCODE to RefSeq and 99.2% from RefSeq to GENCODE (Fig. 6*A*, Supplemental Table S2). The reannotation process eliminated only a very small number of both target and decoy PSMs; therefore, PSM FDRs were maintained at a comparable level. For example, after converting the GENCODE-based search results to RefSeq annotation, the PSM FDR changed from 0.47% to 0.44%. It is worth noting that the reannotation process does not introduce new peptide identifications. Switching from a conservative database (*e.g.* RefSeq) to a comprehensive database (*e.g.* GENCODE) can usually maintain the original identifications, but switching in the reverse direction would eliminate identifications that are supported only by the comprehensive database.

##### Proteomics Data Integration

In a typical proteomics analysis pipeline, MS/MS spectra were first searched against a database to identify PSMs, and then protein identification and quantification were derived based on the PSMs. Integration of different datasets occurs either at the PSM level or the protein level. Using genomic sequence as a common reference, *proBAM* provides a natural solution for proteomics data integration at the PSM level, thus avoiding the problems such as incomparable protein identifiers (IDs). We used four CRC cell lines that were profiled in both the TUM_NCI_60 and the VU_CRC_10 datasets to demonstrate the utility of *proBAM*-based proteomics data integration. The TUM_NCI_60 dataset was searched against the GENCODE protein database, whereas the VU_CRC_10 dataset was searched against customized ENSEMBL66 databases derived from matched RNA-Seq data. After combining individual *proBAM* files into a single *proBAM* file, PSMs were reannotated based on the GENCODE annotation. R scripts for data integration including the running time for each step can be found in Supplementary File 2, using cell line COLO205 as an example. As shown in Fig. 6*B*, the numbers of identified protein and gene groups were notably increased for both COLO205 and HT29. Similar results were obtained for HCT116 and HCT15 cell lines (Supplementary Note and Supplemental Table S3). Importantly, the integration dramatically increased the number of distinct peptides and coding region coverage compared with results for individual datasets (Fig. 6*B* and Supplemental Tables S4 and S5). Integration also added additional unique peptides to a significant number of single-hit genes and proteins in individual datasets, leading to more confident identification of these genes and proteins (Fig. 6*B*). The big increase in the number of distinct peptides and coding region coverage may reflect both the limited coverage of individual experiments and the difference in sample preparation protocols. Specifically, for the TUM_NCI_60 dataset, proteins were first separated by gel electrophoresis and then digested in-gel, whereas for the VU_CRC_10 dataset, protein lysate was first digested and the resulting peptides then were separated by narrow isoelectric point range isoelectric focusing. The first dataset identified a smaller number of proteins but more distinct peptides for individual proteins, whereas the second dataset identified a larger number of proteins with fewer distinct peptides for individual proteins. Thus, *proBAMsuite*-enabled proteomics data integration may leverage existing datasets to gain a more comprehensive coverage of a proteome of interest.

This approach easily can be extended to the integration of more than two datasets. However, an important consideration when applying this approach to the integration of data from a large number of samples is the appropriate control of protein- and gene-level FDRs, which may quickly become uncontrolled with increased sample size (8, 31). Because the PSMs of decoy peptides are kept in *proBAM* files and are included in protein and gene inference, *proBAMtools* can provide protein- and gene-level FDRs for an integrated dataset using the target-decoy strategy. In the TUM_NCI_60 dataset, with a PSM FDR threshold of 1%, the protein- and gene-level FDRs increased linearly with sample size and reached 17% and 19%, respectively, when all 60 samples were combined (Fig. 6*C*). Both the protein- and gene-level FDRs were less than 0.9% for each sample. This problem can be alleviated by applying a more stringent PSM FDR (Fig. 6*C*). In contrast to its dramatic impact on protein- and gene-level FDRs, strengthening the PSM FDR had only a relatively moderate impact on the numbers of protein and gene identifications (Fig. 6*D*).

With the ability to integrate multiple proteomics datasets while controlling protein- and gene-level FDRs, *proBAMsuite* can facilitate more comprehensive proteomic validation of novel coding genes and transcripts predicted by genomics projects. In the GENCODE annotation provided by the ENCODE project, transcripts are categorized as known, novel, or putative, reflecting their similarity to preexisting models in EntrezGene or Swissprot/Uniprot (34). By integrating data from all 60 cell lines in the TUM_NCI_60 dataset, with a protein-level FDR of 4.5% (PSM FDR 0.5%), we identified a total of 11,631 protein groups, among which 272 and 617 could only be explained by novel or putative transcripts, respectively (Supplementary Note and Supplemental Fig. S6). Similarly, for GENCODE transcripts classified by an alternative scheme with three confidence levels, we identified 410 protein groups that could only be explained by automated annotated transcripts (Supplementary Note and Supplemental Fig. S6). These results demonstrate the potential of proteomics data in consolidating genome annotation and suggest that full realization of this potential would benefit from *proBAM*-based integration of a large number of existing proteomics datasets.

##### Proteogenomic Data Integration

**Fig. 7.**
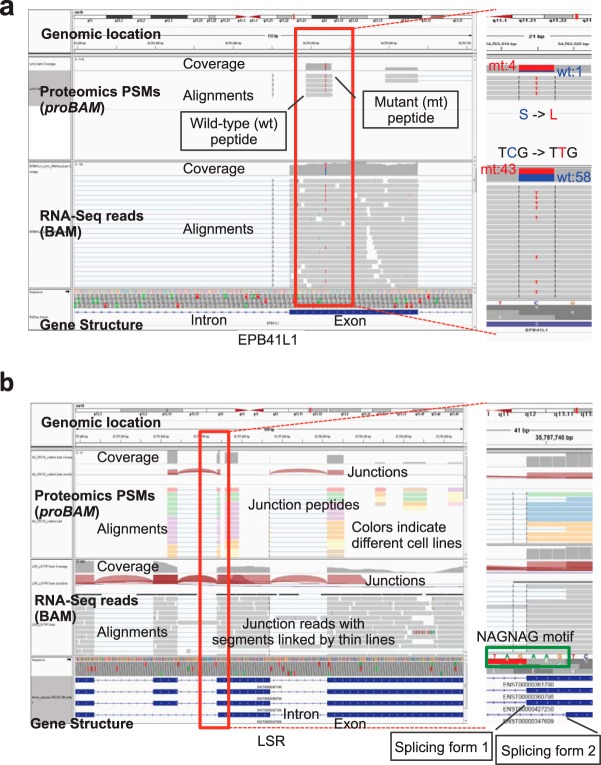
**Proteogenomics data integration.** (*A*) Integrative Genomics Viewer (IGV) snapshot of a heterozygous Ser13Leu mutation of gene *EPB41L1* (inside the red box) in the LoVo cell line. The ratio between wild-type and mutant alleles is skewed toward mutant in the proteomics data (*upper panel*) but not the RNA-Seq data (*lower panel*). (*B*) Integrative Genomics Viewer (IGV) snapshot of a tandem splicing site in gene *LSR* (inside the red box), which was confirmed by both RNA-Seq (*lower panel*) and proteomics (*upper panel*) data from multiple cell lines as indicated by different colors.

Using genome sequence as a common reference, *proBAM* also facilitates seamless inference across proteomic and genomic or transcriptomic datasets. We demonstrate this with three examples from the VU_CRC_10 dataset, which was searched against customized protein databases built from matched RNA-Seq data (24, 26). In the first example, we investigated coding single nucleotide variants at both the RNA and protein levels simultaneously. Figure 7*A* shows a heterozygous Ser13Leu mutation of EPB41L1 in the LoVo cell line. We previously confirmed this mutant peptide by targeted analysis of tumor lysates spiked with synthetic, isotope-labeled peptide standards (24). Interestingly, the expression ratio between mutant and wild-type alleles is skewed toward mutant at the protein level (4:1) but not at the RNA level (43:58), suggesting either preferential translation of the mutant allele transcript or higher stability of the protein product resulting from the mutated allele (*p* = 0.05, one-sided z-test). Thus, *proBAM* allows researchers to verify mutations detected at the genomic level with proteomic evidence and to compare allele expression patterns between proteomics and transcriptomics data.

The second example demonstrates the proteomic validation of alternative splicing isoforms. About 30% of human genes contain introns ending in NAGNAG (N represents any nucleotide), where alternative splicing can create transcript isoforms by inclusion or exclusion of three bases; however, only some of these motifs are functional (35). The NAGNAG motif was found in a splice acceptor in the gene *LSR* (Fig. 7*B*). RNA-Seq data support the existence of tandem splicing at this site and proteomics data further prove that both splicing isoforms are translated into proteins in multiple cell lines and thus are very likely to be functional (Fig. 7*B*). The last example shows how *proBAM* facilitates the proteomic validation of novel coding regions predicted by RNA-Seq. As shown in Supplemental Fig. S7, peptides were identified from multiple samples for a novel exon region predicted by RNA-Seq data, which is located near *EPB41*.

## DISCUSSION

We have developed *proBAMsuite*, a bioinformatics framework for genome-based representation and analysis of proteomics data. In contrast to existing proteomics data formats that use proteins as the data organization unit, the *proBAM* format organizes proteomic data based on the corresponding genome, thereby providing a fundamentally different way to structure and reference proteomics data. This new data format and the associated *proBAMtools* address the inherent limitations of the existing protein-centric data formats and data analysis tools. First, the interpretation of proteomics data is significantly enhanced with the rich genomic annotation information. Second, PSMs can be easily reannotated using user-specified gene annotation schemes and assembled into both protein and gene identifications. Third, using the genome as a common reference, *proBAMsuite* facilitates seamless proteomics and proteogenomics data integration. Finally, *proBAM* files can be visualized readily in genome browsers and thus are immediately available to a general audience beyond the proteomics community.

The *proBAM* format offers a few unique features compared with other related approaches (14–19). First, *proBAM* holds more information in one file. In addition to amino acid sequences, *proBAM* also includes information on sequence variants, the relationship between peptides and exon annotation (*e.g.* exon–exon junction peptides and within exon peptides), PSM scores, and peptide charge states, among others. Second, *proBAM* is highly analogous to BAM, which allows it to link proteomics data to a large number of bioinformatics tools and methods that have already been developed for the analysis and visualization of BAM files. Third, *proBAM* stores data in a compact format, and the data volume reduction enables more effective data exchange. Finally, the *proBAM* format is supported by the *proBAMsuite*, which provides tools for file conversion and downstream data analysis, enabling easy adoption and use of the *proBAM* format for any proteomics laboratories.

By effectively leveraging genomic annotation information, the *proBAM*-driven approach reveals useful insights that would not be apparent from analyses based on existing protein-centric data formats. For example, it enables easy identification of junction peptides or peptides specific to a gene or protein for targeted proteomics analysis. It can also distinguish peptides mapping to multiple genomic locations from those mapping to a unique location. Although a large number of shared peptides are typically observed in proteomics experiments, the *proBAM*-based analysis showed that more than 90% of the peptides were mapped to a unique genomic location. This observation motivated the implementation of gene-level assembly in *proBAMtools*, which reduces the ambiguity in peptide assembly and improves the reliability of spectral-counting-based quantification. Although not investigated in this paper, the gene-level assembly may also improve the reliability of labeled proteomics quantification because of the reduced ambiguity in peptide assignment.

Although recent proteomics studies have started to report gene-level inference (36), it is typically achieved by associating peptides with genes through their proteins based on ID mapping tables. The *proBAM*-driven approach is the only one that directly uses the genomic location of PSMs and assigns peptides to proteins or genes based on the Range infrastructure developed by the genomics community (23). Therefore, it could distinguish gene- and protein-specific peptides from those mapped to multiple proteins or genes. Spectral count data are generated at both gene and protein levels independently, avoiding the complication in converting protein-level counts into gene level. The *proBAM* framework for protein and gene inference is generic and can be extended in the future to include additional data such as intensity-based peptide mass from MaxQuant (37) to improve quantification.

Using the genome as a common reference, *proBAM* meets critical needs in proteomics and proteogenomics data integration. *proBAM* alleviates the compatibility problem by bringing different proteomics datasets into the same coordinate system, *i.e.* the genome, thereby allowing data integration without re-searching the raw data. When different versions of the reference genome are used, tools developed in the genomics field for converting genome coordinates between assemblies in BAM/SAM format, such as CrossMap (38), could be directly applied to the *proBAM* files. *proBAM* also facilitates seamless integration between proteomics data and genomic or transcriptomic data, allowing cross-referencing and consolidating novel discoveries in proteogenomic studies, including genomic mutations and allele expression patterns, predicted splicing isoforms, and novel coding regions. We show that integrating proteomics data from two independent cell line studies almost doubled the number of distinct peptides and coding region coverage for all four cell lines. Integrating all three datasets used in the study achieved an overall coverage of 18% for the whole human coding genome. We show that applying a stringent PSM FDR can effectively control protein- and gene-level FDRs when integrating data from a large number of samples. Although this strategy only moderately reduces the numbers of identified proteins and genes, it dramatically reduces the number of identifiable spectra. In the TUM_NCI_60 dataset, the total spectra count dropped from 3,410,633 to 2,242,690 when the PSM FDR was reduced from 1% to 0.1%. Previously, we used a second step to rescue high-quality PSMs that were excluded by the stringent PSM FDR threshold (*i.e.* to relax the PSM FDR threshold to 1% for the confidently identified proteins) (8). Applying the same procedure to the TUM_NCI_60 dataset increased the total number of identifiable spectra to 3,390,728.

We expect that many researchers would benefit from the proteomics datasets made available in the JBrowse (39)-based genome browser, which allows visualizing all PSMs against the human genome. An important future work is to allow hyperlinking each PSM to a visual display of the associated spectrum, which is possible because *proBAM* preserves the link to supporting spectrum for each PSM, and JBrowse is an open source project. The *proBAM* files can also be downloaded and visualized in the more flexible desktop-based Integrative Genomics Viewer (40). Moreover, toolkits such as the BEDTools (41) can be used to convert *proBAM* files to other file formats, such as the Browser Extensible Data (BED) format, which can be uploaded to the UCSC Genome Browser or Galaxy.

In conclusion, we have developed *proBAM* as a new format for proteomics data, and this study establishes *proBAMsuite* as a useful bioinformatics framework for proteomics and proteogenomics research.

## Supplementary Material

Supplemental Data
