# Supplementary material for: proBAMsuite, a Bioinformatics Framework for Genome-Based Representation and Analysis of Proteomics Data
Source: Mol Cell Proteomics. 2015 Dec 11;15(3):1164–75. doi: 10.1074/mcp.M115.052860 (PMC4813696; doi:10.1074/mcp.M115.052860)
Supplement: Supplemental Data [file supp_15_3_1164__index.html]

proBAMsuite, a bioinformatics framework for genome-based representation and analysis of proteomics data — proBAMsuite, a Bioinformatics Framework for Genome-Based Representation and Analysis of Proteomics Data — proBAMsuite: Genome-Based Analysis of Proteomics Data — Supplemental Data 

# proBAMsuite, a Bioinformatics Framework for Genome-Based Representation and Analysis of Proteomics Data

## Supplemental Data

- Supplemental Note, Figures, and Tables (.pdf, 2.5 MB) - Supplementary Note Supplementary Figure 1. A computational pipeline to generate and analyze proBAM files. Supplementary Figure 2. proBAM makes proteomics identifications interchangeable between different gene annotation schemes. Supplementary Figure 3. Screenshots from the proteogenomics browser Supplementary Figure 4. Chromosome CDS coverage of the TUM\_NCI\_60 dataset Supplementary Figure 5. Gene CDS coverage analysis results for the TUM\_NCI\_60 dataset Supplementary Figure 6. Proteomic validation of GENCODE transcripts using the TUM\_NCI\_60 dataset Supplementary Figure 7. IGV snapshot of a novel coding region predicted by RNA-Seq data (middle panel) and verified by proteomics data from four cell lines as indicated by different colors (top panel) Supplementary Table 1. Definition of the proBAM format. Supplementary Table 2. Detailed proteomics identification data from the TUM\_NCI\_60 dataset Supplementary Table 3. The numbers of identified gene and protein groups from four colorectal cancer cell lines in the TUM\_NCI\_60 and VU\_CRC\_10 datasets separately or in combination. Supplementary Table 4. The genome CDS coverage of four colorectal cancer cell lines in the TUM\_NCI\_60 and VU\_CRC\_10 datasets separately or in combination. Supplementary Table 5. The numbers of distinct peptides identified from four colorectal cancer cell lines in the TUM\_NCI\_60 and VU\_CRC\_10 datasets separately or in combination.
- Supplementary File 1 (.pdf, 619 KB) - A computational pipeline to generate and analyze proBAM files.
- Supplementary File 2 (.pdf, 37 KB) - Example R code of proBAM-based data integration for cell line COLO205.
