# Supplementary material for: proBAMsuite, a Bioinformatics Framework for Genome-Based Representation and Analysis of Proteomics Data
Source: Mol Cell Proteomics. 2015 Dec 11;15(3):1164–75. doi: 10.1074/mcp.M115.052860 (PMC4813696; doi:10.1074/mcp.M115.052860)
Supplement: Supplemental Data [file 10.1074_M115.052860_mcp.M115.052860-1.pdf]

proBAMsuite, a bioinformatics framework for genome-based representation and analysis of proteomics data

Xiaojing Wang[1], Robbert J. C. Slebos[2,3], Matthew C. Chambers[1], David L. Tabb[1,2], Daniel C. Liebler[1,2,3], Bing Zhang*[1,4]

SUPPLEMENTARY DATA

Supplementary Note

Supplementary Figures

Supplementary Figure 1. A computational pipeline to generate and analyze *proBAM* files.

Supplementary Figure 2. *proBAM* makes proteomics identifications interchangeable between different gene annotation schemes.

Supplementary Figure 3. Screenshots from the proteogenomics browser

Supplementary Figure 4. Chromosome CDS coverage of the TUM_NCI_60 dataset

Supplementary Figure 5. Gene CDS coverage analysis results for the TUM_NCI_60 dataset

Supplementary Figure 6. Proteomic validation of GENCODE transcripts using the TUM_NCI_60 dataset

Supplementary Figure 7. IGV snapshot of a novel coding region predicted by RNA-Seq data (middle panel) and verified by proteomics data from four cell lines as indicated by different colors (top panel)

Supplementary Tables

Supplementary Table 1. Definition of the *proBAM* format.

Supplementary Table 2. Detailed proteomics identification data from the TUM_NCI_60 dataset

Supplementary Table 3. The numbers of identified gene and protein groups from four colorectal cancer cell lines in the TUM_NCI_60 and VU_CRC_10 datasets separately or in combination.

Supplementary Table 4. The genome CDS coverage of four colorectal cancer cell lines in the TUM_NCI_60 and VU_CRC_10 datasets separately or in combination.

Supplementary Table 5. The numbers of distinct peptides identified from four colorectal cancer cell lines in the TUM_NCI_60 and VU_CRC_10 datasets separately or in combination.

Supplementary Files

Supplementary File 1. A computational pipeline to generate and analyze *proBAM* files.

Supplementary File 2. Example R code of *proBAM*-based data integration for cell line COLO205.

SUPPLEMENTARY NOTE

CDS coverage analysis

We carried out a sequence coverage analysis using the TUM_NCI_60 dataset to explore whether the coding sequence (CDS) coverage approaches saturation with data from a large number of cell lines of diverse tissue origin. Specifically, we used down-sampling to study how the CDS coverage or the total numbers of identified genes or proteins increase with sample size by repeating the analysis on random subsets of the samples from size 1 to 61.

For genome level CDS coverage, the coverage ratio increased almost linearly with the sample size, indicating that the proteome sequence coverage is far from reaching a plateau (**Fig. 4c**). The increase of the total numbers of identified protein and gene group (after parsimony algorithm, support of at least two peptides and at least one discriminative peptide) slowed slightly at sample sizes above 10, but did not reach saturation for this dataset (**Fig. 6d)**.

With increasing sample sizes, the numbers of genes with different degrees of sequence coverage changed in different patterns (**Supplementary Fig. S5**). The number of genes with CDS coverage from 5% to 20% increased almost linearly, suggesting that genes with relatively low expression level benefit most from increased sample size. The number of genes with CDS coverage greater than 50% increased very slowly; these are likely to be genes coding for abundant and easily detectable proteins. Interestingly, the number of genes with very low proteomics coverage (0~5%) decreased with increasing sample size, suggesting that combining proteomics datasets increases not only the power to detect more genes/proteins, but also improves sequence coverage of the identified genes.

proBAM facilitates this type of analysis by providing an easily computable file format. Functions for calculating sequence coverage at the whole genome or individual gene levels based on *proBAM* files are available in the *proBAMtools*.

***proBAM* facilitates genome-based proteomics data interpretation.**

Sixty-one *proBAM* files were generated from the TUM_NCI_60 dataset searched against the GENCODE protein database. Merging all *proBAM* files yielded 4,535,568 PSGMs (Peptide-Spectrum-Genomic location-Match), 3,410,633 identifiable spectra, 3,456,743 PSMs, and 141,834 unique peptides. Here we used three features, including peptide sequence, charge, and modification, to define a unique peptide. If we only consider unique peptide sequences, the number decreased to 113,534 (**Supplementary Table S2**).

With the genomic sequence mapping information available in the *proBAM* files, we can easily identify genomic regions from which the peptides were derived. We found 106,852 of the 113,534 peptides (94.11%) mapped to unique locations. Among these peptides, 79,288 (74.20%) were mapped to exon regions and 27,564 (25.80%) were mapped to exon-exon junction regions (**Supplementary Table S2**). The exon-exon junction peptides provide direct evidence for isoform structures. The majority of the exon-exon junction peptides spanned two exons (26,851 out of 27,564, 97.41%), whereas a small number spanned more than two exons (e.g., 662 involved 3 exons).

The rest of the peptides (6,682, 5.89%) were mapped to more than one genomic location, most of which were mapped to 2 locations (4,724, 4.16%) (**Supplementary Table S2**). Very few peptides mapped to more than 2 locations (1,114 peptides mapped to 3 locations; 784 peptides

mapped to 4-10 locations, and only 60 peptides mapped to more than 10 locations). Most of these peptides come from conserved gene families, such as the *ZNF*, *HIST* and *PCDHB* families.

Genome-based proteomics data integration

Using the three-step procedure described in **Methods** and illustrated in **Supplementary Fig. S2a**, we switched the gene annotation scheme for the TUM_NCI_60 dataset searched against the GENCODE database to RefSeq database. The proteomics identification results after each step are summarized in **Supplementary Fig. S2b**. After the three-step procedure, the numbers of retained proteomics identifications were very close to those generated by a direct search against the RefSeq database (**Fig. 6a and Supplementary Table S2**). This result demonstrates the ability of *proBAM* in facilitating switching between different gene annotation schemes without new database searching. Accordingly, proteomics datasets generated by searching against different databases can be mapped to genome for integration and then applied to the same gene annotation scheme for integrative analysis.

There are four common CRC cell lines in the TUM_NCI_60 and the VU_CRC_10 datasets, including COLO205, HCT116, HCT15, and HT29. We used data from these cell lines to demonstrate *proBAM*-based proteomics data integration. As the TUM_NCI_60 and the VU_CRC_10 datasets were searched against the GENCODE v19 database and the customized ENSEMBL66 database, respectively, we first filtered the *proBAM* from VU_CRC_10 dataset to fulfill the GENCODE v19 annotation scheme. **Supplementary Table S3** summarizes the number of protein/gene group after parsimony from the four cell lines based on individual datasets as well as from the combined dataset, where a protein/gene group is supported by at least two peptides and at least one discriminative peptide.

Using data from all four cell lines, we investigated the impact of data integration on genome CDS region coverage, the numbers of distinct peptides, and identified protein and gene groups. For each cell line, the combined CDS coverage ratio was almost double the ratios in individual datasets (**Supplementary Table S4 and Figure 6b**), suggesting that data integration significantly improved the CDS coverage for all four cell lines. Similar results were found for the number of distinct peptides. Using cell line COLO205 as an example, after switching to GENCODE annotation, we found 25,427 distinct peptides in the VU_CRC_10 dataset and 23,570 in the TUM_NCI_60 dataset. After the integration, we found 42, 983 distinct peptides, which almost doubled the counts in individual datasets (**Supplementary Table S5 and Fig. 6b**). Data integration also increased the number of gene and protein groups (**Fig. 6b and Supplementary Table S3**). For example, for the cell line HT29, we identified 4,139 and 2,591 gene groups in the VU_CRC_10 and the TUM_NCI_60 datasets, respectively, and integrating the datasets increased the number to 4,906. We noticed that a higher increase was observed for the CDS coverage and the number of distinct peptides compared to the number of gene and protein groups, suggesting individual studies may sample different protein sequences even for similar protein pools. Moreover, the number of protein groups with unique proteins is much lower than that of gene groups with unique genes (**Supplementary Table S3**), indicating insufficient sequence coverage in proteomics data for distinguishing protein isoforms and highlighting the value of integrating data from multiple studies.

Interestingly, some peptides labeled as novel junction peptides in the VU_CRC_10 dataset searched against RNA-Seq data-derived customized ENSEMBL66 databases were retained after converting to the GENCODE v19 annotation. These peptides mapped to regions un-annotated in ENSEMBL66 that were annotated in the more recent GENCODE annotation. This, to some

extent, demonstrates the value of RNA-Seq data-derived customized databases in proteomics database search. Meanwhile, *proBAM* facilitates co-visualization of genomic and proteomic data in genome browsers to provide visual confirmation of novel alternative splicing or new genes predicted by RNA-Seq using proteomics data (**Supplementary Fig. S7**).

Verifying protein-coding transcripts in GENCODE using the TUM_NCI_60 dataset

a. Known, Novel and Putative transcripts

Proteomics data can be used to verify genome annotation. The GENCODE project provides three classifications for transcripts based on the strength of supporting evidence. The status reflects the similarity between the annotated CDS and a pre-existing model in Entrez Gene or Swissprot / Uniprot (assuming one can be found)(1). In particular, a 'known' CDS refers to a sequence that can be mapped with 100% identity to a known protein sequence from RefSeq, Swissprot or Uniprot. A 'novel' CDS shares at least 60% identity in length with a known CDS. A 'putative' CDS has less than 60% identity with a known CDS.

We mapped all the peptides to the three classes. Since the three different classifications are defined at transcript level, it is conceivable that peptides map to multiple classes, as shown in **Supplementary Fig. S6a**. Among the 126,832 identified peptides at PSM FDR 0.5%, 124,804 (98.4%) overlapped with known transcripts CDS, 36,762 (29.0%) overlapped with novel transcripts CDS and 38,263 (30.2%) overlapped with putative transcripts CDS. However, only a small proportion of peptides uniquely overlapped with novel (556, 0.44%) and putative (1,352, 1.07%) transcripts CDS.

Next, we sought to find the numbers of transcripts with proteomic evidence in each class. Because transcripts in different classes share exons, we used a priority strategy to map the proteomics data to CDSs in the three classifications. First, all peptides were mapped against the known transcripts; next, the remaining peptides were mapped against the novel transcripts; finally, unmapped peptides were mapped against the putative transcripts. As a result, 46,011 (68.45%) known transcripts had peptide evidence, whereas the numbers of novel and putative transcripts with peptide evidence were 508 (4.97%) and 1,101 (6.14%), respectively (**Supplementary Fig. S6b**).

We then assigned identified protein groups (after parsimony) to known, novel and putative classifications. First, we determined whether any item in a protein group belongs to known transcripts; if so, the protein group was classified as known. We then checked whether any item in the remaining protein groups belonged to novel transcripts; the remaining protein groups were labeled as putative (**Supplementary Fig. S6b**). As a result, 272 and 617 protein groups were classified as novel and putative, respectively.

b. Level 1, 2 and 3 transcripts

Similarly, we also used proteomics data to validate GENCODE transcripts classified by an alternative scheme with three confidence levels (2). Level 1 refers to validated transcripts, level 2 includes manually annotated transcripts, and level 3 includes automated annotated transcripts. The CDS numbers at each level are summarized in **Supplementary Fig. S6c**. We found 21,880 (17.3%), 124,316 (98.0%) and 43,677 (34.4%) peptides overlapped with level 1, 2, 3 transcript CDS regions respectively. Among the 43,677 level 3 peptides supported by the automated

annotation pipeline, only 1,321 could be uniquely associated with the level 3 transcripts (**Supplementary Fig. S6c)**.

By applying the same method mentioned above, we found peptide evidence for 4,073 (62.6%) level 1 transcripts, 45,277 (57.8%) level 2 peptides, and 651 (6.15%) level 3 peptides **(Supplementary Fig. S6d)**. The numbers of protein groups were 850, 10,371 and 410 for level 1, 2, 3 transcripts respectively **(Supplementary Fig. S6d)**. Both validated and manually annotated transcripts had similar levels of support from proteomic evidence, suggesting a high quality of the manually annotated transcripts. Interestingly, despite a much lower overall validation ratio for the automated annotated transcripts, some of these were supported by peptide evidence, demonstrating the potential of proteomics data in improving genome annotation.

Despite a much lower overall ratio for the novel, putative, and automated annotated protein groups, these results demonstrate the potential of proteomics data in consolidating genome annotation, and suggest that full realization of this potential would benefit from *proBAM*-based integration of a large number of existing proteomics datasets.

References:

1. Ezkurdia, I., del Pozo, A., Frankish, A., Rodriguez, J. M., Harrow, J., Ashman, K., Valencia, A., and Tress, M. L. (2012) Comparative proteomics reveals a significant bias toward alternative protein isoforms with conserved structure and function. *Mol Biol Evol* 29, 2265-2283

2. Harrow, J., Frankish, A., Gonzalez, J. M., Tapanari, E., Diekhans, M., Kokocinski, F., Aken, B. L., Barrell, D., Zadissa, A., Searle, S., Barnes, I., Bignell, A., Boychenko, V., Hunt, T., Kay, M., Mukherjee, G., Rajan, J., Despacio-Reyes, G., Saunders, G., Steward, C., Harte, R., Lin, M., Howald, C., Tanzer, A., Derrien, T., Chrast, J., Walters, N., Balasubramanian, S., Pei, B., Tress, M., Rodriguez, J. M., Ezkurdia, I., van Baren,

J., Brent, M., Haussler, D., Kellis, M., Valencia, A., Reymond, A., Gerstein, M., Guigo, R., and Hubbard, T. J. (2012) GENCODE: the reference human genome annotation for The ENCODE Project. *Genome Res* 22, 1760-1774

Supplementary Figure 1. A computational pipeline to generate and analyze *proBAM* files. An R package, *pepXMLTab*, is used to convert the pepXML files to a tabular format, which includes all confident peptide-spectrum matches (PSMs) in the pepXMLs. The second R package, *proBAMr*, maps all normal, variant, and novel junction peptides in the PSMs to the genome and stores the mapping information in a modified SAM format (*proSAM*), which is then converted to *proBAM* files using the SAMtools. The third package *proBAMtools* provides functions to facilitate genome-based data interpretation, peptide assembly, and data integration.

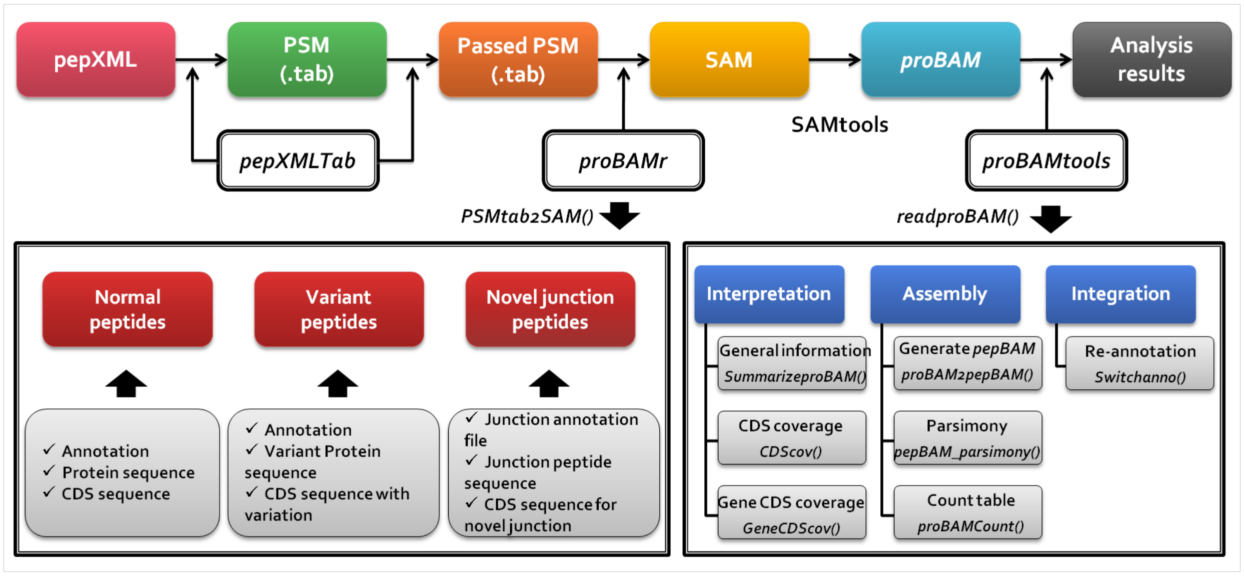

Supplementary Figure 2. *proBAM* makes proteomics identifications interchangeable between different gene annotation schemes. (a) A three-step procedure to re-annotate *proBAM* files according to the specified gene annotation scheme. **(b)** Results for annotation switching from GENCODE to Refseq for the TUM_NCI_60 dataset. The table lists spectral and peptide counts summarized from the input *proBAM* file and the results after each step as illustrated in (**a**). The results were generated at PSM FDR 1%.

a

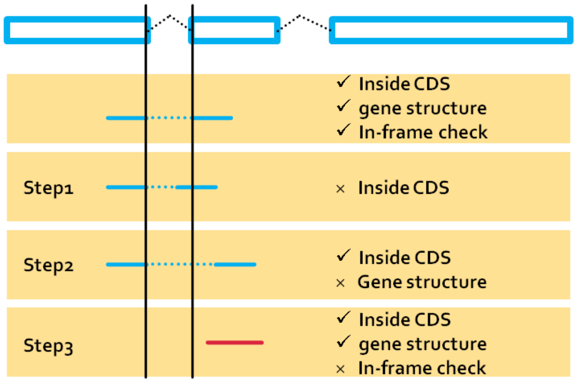

b

Total rows	Spectra	PSM	Distinct peptide	Distinct peptide sequence	Unique location peptide
4535568	3410633	3456743	141834	113534	106852
4471795	3398976	3444704	138565	110409	104386
4461509	3397893	3443188	137955	109843	103988
4426805	3373025	3418131	137126	109172	103610

Supplementary Figure 3. Screenshots from the proteogenomics browser. (a) Alignment of all variant peptides in the CPTAC_CRC dataset to chromosome 12. (**b)** Zoomed view of the KRAS locus with isoform structures showing in the bottom track and proteomics data in the other tracks. In the CPTAC_CRC dataset, a variant peptide was found in the first exon of KRAS (first track), and peptide evidence was observed for three exons and one junction, with alignment data showing in the second track and coverage data showing in the third track. In the VU_CRC_10 dataset, peptide evidence was observed for all five exons in the two KRAS isoforms as well as two junctions (the 4th to 6th tracks). (**c)** Further expanded view of the first KRAS exon region. Peptide evidence was observed for the well-known, hotspot mutations in KRAS in both the CPTAC_CRC (tumors) and the VU_CRC_10 (cell lines) datasets. **(d)** Detailed information retrieved and displayed for one of the peptide-spectrum matches (PSMs).

a

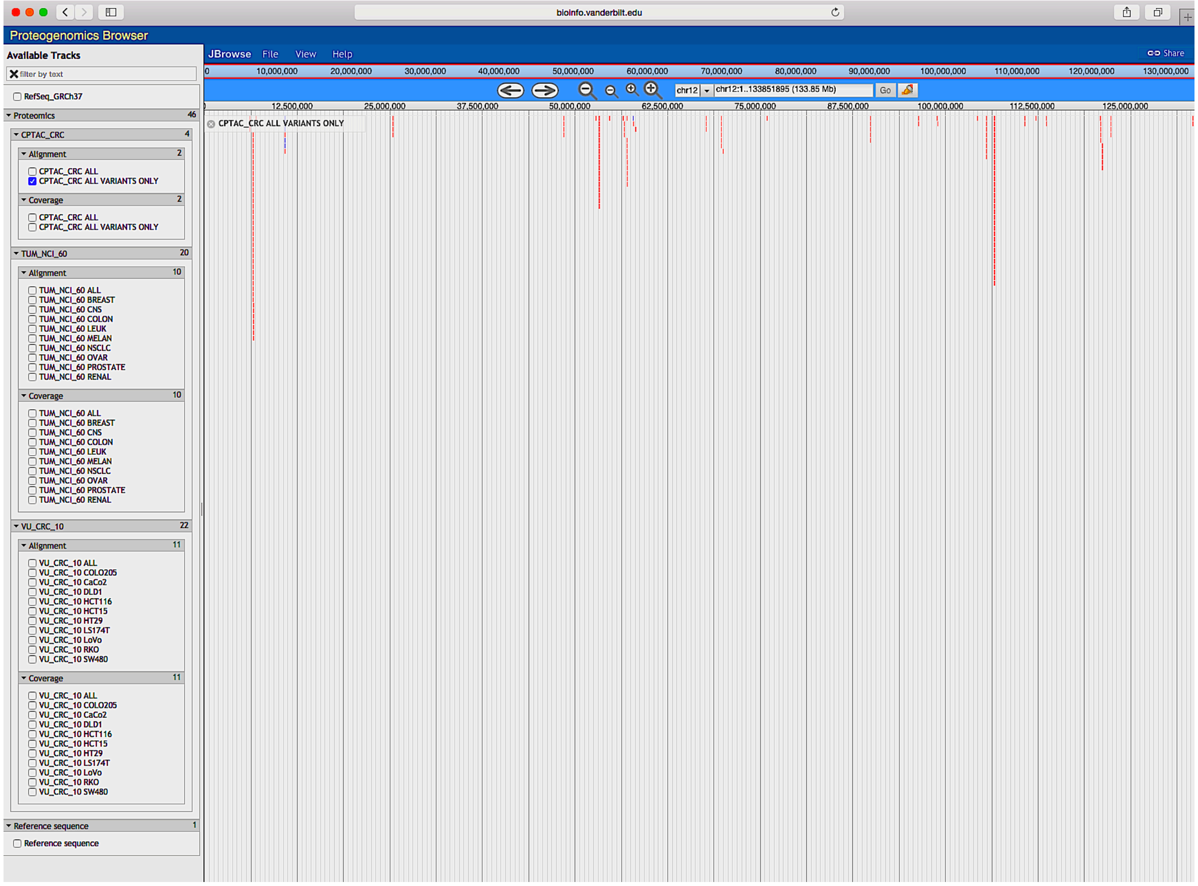

b

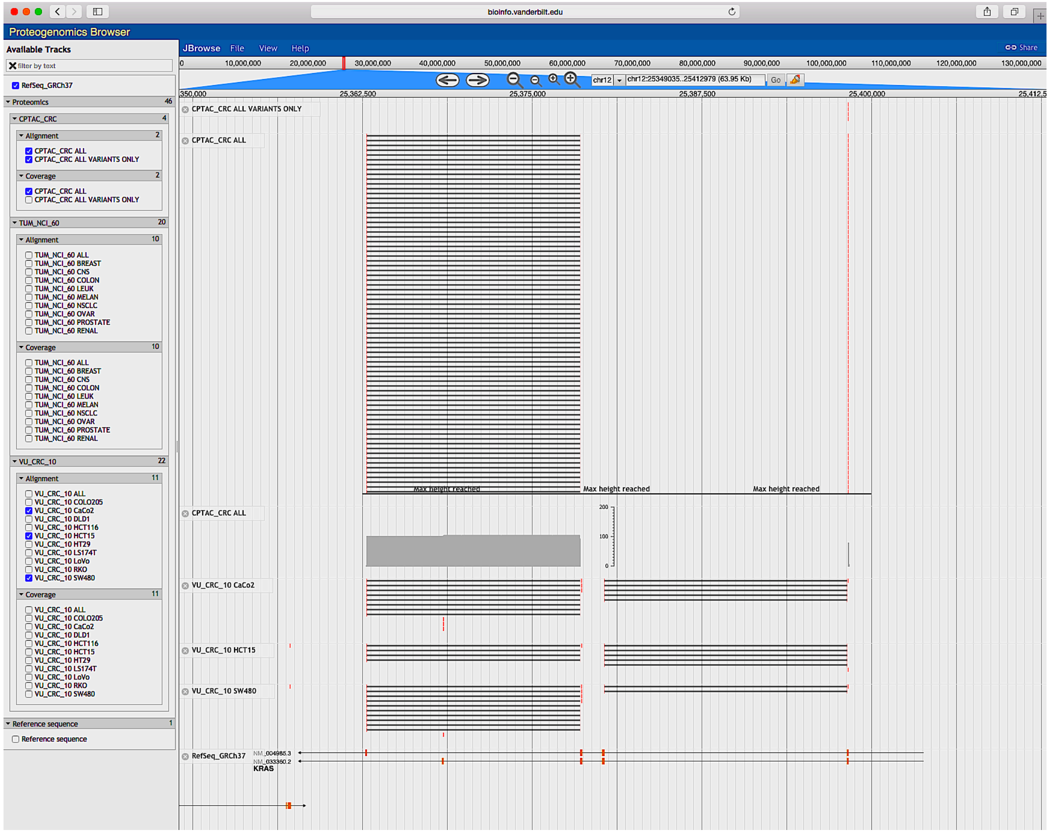

bioinfo.vanderbilt.edu
Proteogenomics Browser
Available Tracks
JBrowse File View Help
RefSeq_GRCh37
Proteomics
CPTAC_CRC
Alignment
CPTAC_CRC ALL
CPTAC_CRC ALL VARIANTS ONLY
Coverage
TUM_NCI_60
VU_CRC_10
Reference sequence
CPTAC_CRC ALL VARIANTS ONLY
CPTAC_CRC ALL
Max height reached
VU_CRC_10 CaCo2
VU_CRC_10 HCT15
VU_CRC_10 SW480
RefSeq_GRCh37
KRAS

c

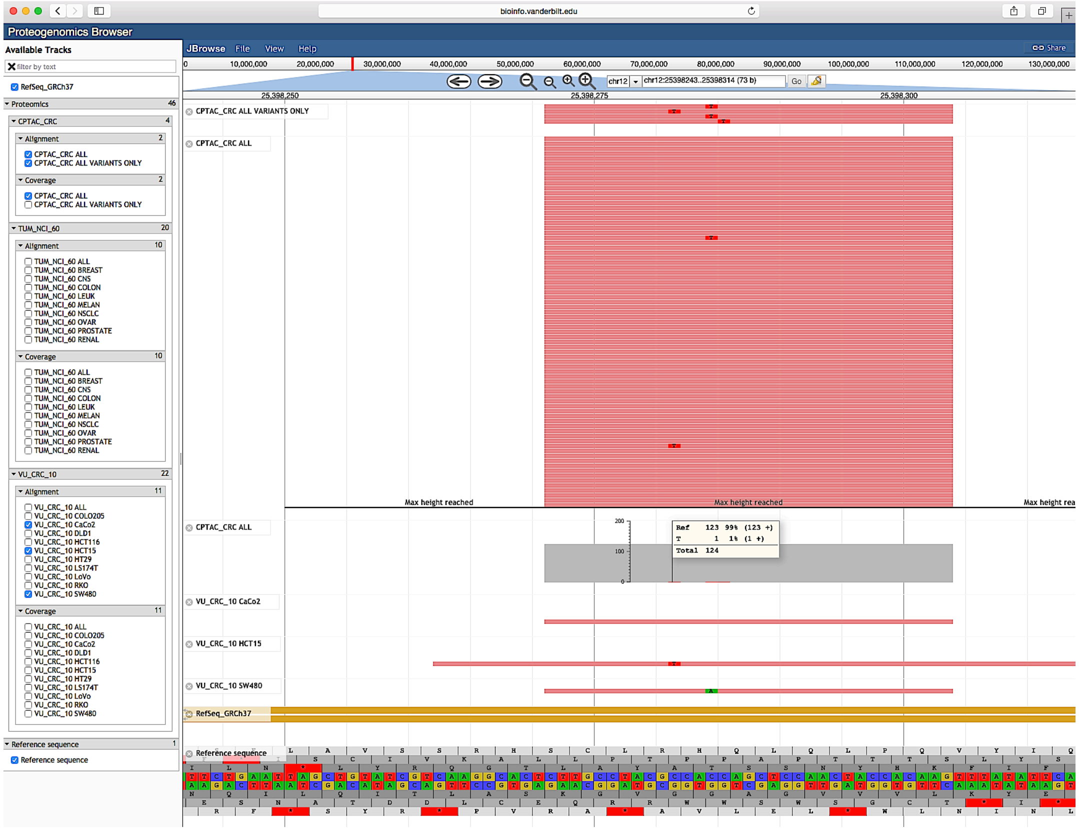

bioinfo.vanderbilt.edu
Proteogenomics Browser
Available Tracks
JBrowse File View Help
RefSeq_GRCh37
Proteomics
CPTAC_CRC
TUM_NCI_60
VU_CRC_10
CPTAC_CRC ALL VARIANTS ONLY
CPTAC_CRC ALL
Max height reached
Ref 123 99% (123 +)
T 1 1% (1 +)
Total 124
VU_CRC_10 CaCo2
VU_CRC_10 HCT15
VU_CRC_10 SW480
RefSeq_GRCh37
Reference sequence

d

match W_VU_20120829_A0218_2J_R_FR04.0.1.4215

**Name**	W_VU_20120829_A0218_2J_R_FR04.0.1.4215
**Type**	match
**Position**	chr12:25398271..25398303 (+)

Sequence and Quality

C	T	T	G	C	C	T	A	C	G	T	C	A	C	C	A	G	C	T
255	255	255	255	255	255	255	255	255	255	255	255	255	255	255	255	255	255	255

C	C	A	A	C	T	A	C	C	A	C	A	A	G	
255	255	255	255	255	255	255	255	255	255	255	255	255	255	=

**CIGAR**	33M
**MD**	10C22
**NH**	1
**NM**	1
**RG**	TCGA-AA-A01R-01A-23
**XA**	0
**XC**	2
**XG**	V
**XL**	1
**XM**	-
**XN**	0
**XP**	LVVVGAGDVGK
**XR**	LVVVGAGGVGK
**XS**	0.0007999999797903001
**XT**	2
**Duplicate**	no
**Length on ref**	33
**Multi segment template**	no
**Qc failed**	no
**Qual**	

255 255 255 255 255 255 255 255 255 255 255 255 255 255 255 255 255 255 255 255 255 255 255 255
255 255 255 255 255 255 255 255 255

**Secondary alignment**	no
**Seq**	

CTTGCCTACGTCACCAGCTCCAACTACCACAAG=

**Seq length**	33
**Seq reverse complemented**	yes
**Source**	ALL.bam
**Supplementary alignment**	no
**Template length**	0
**Unmapped**	no

OK

Supplementary Figure 4. Chromosome CDS coverage of the TUM_NCI_60 dataset. Data for each chromosome is presented in two columns. The pink bands in the left column represent the coding regions in human genome (hg19). The histograms in the right column demonstrate log transformed spectral counts for individual positions. Quantitative coverage data for individual chromosomes are presented in **Fig.4a** and the average coverage was only 12%. The coverage was calculated using peptides with PSM FDR at 1%.

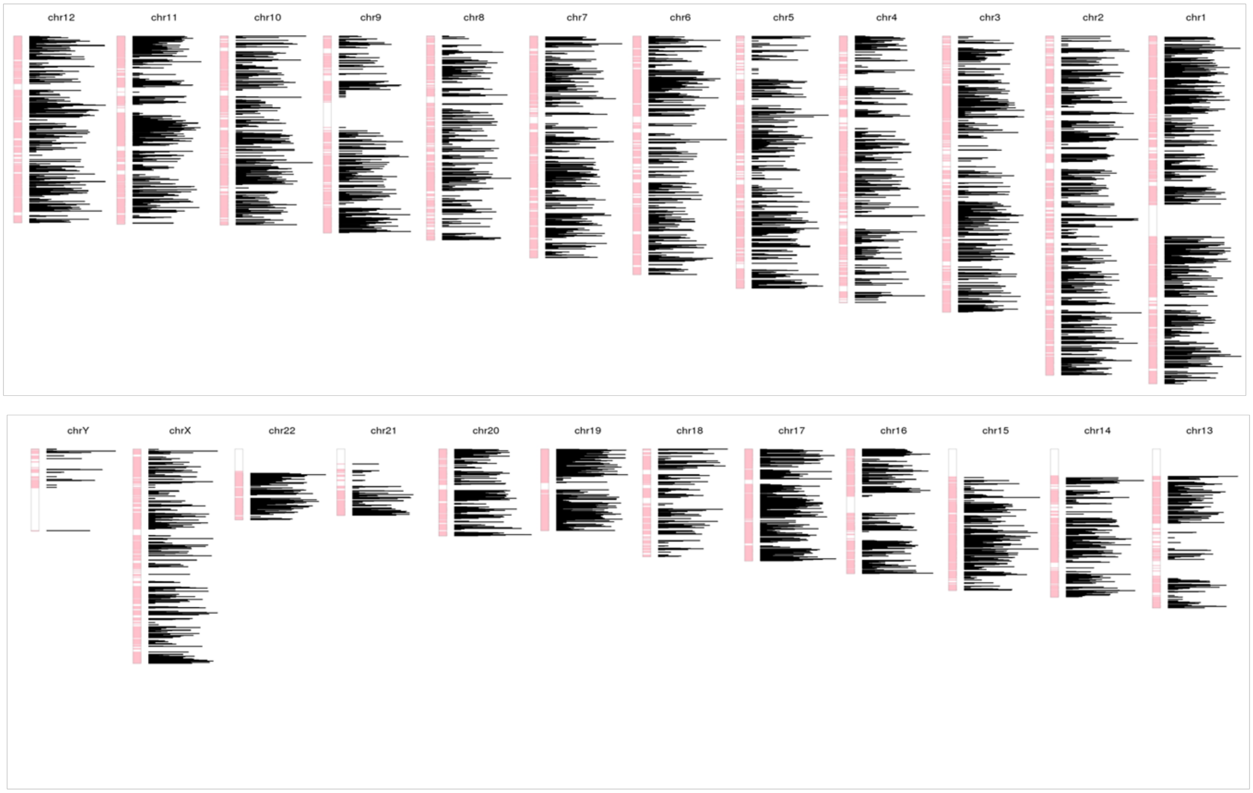

Supplementary Figure 5. Gene CDS coverage analysis results for the TUM_NCI_60 dataset. Cumulative distribution of the number of genes at different coding sequence (CDS) coverage levels as a function of sample size. The results were generated at PSM FDR 1%.

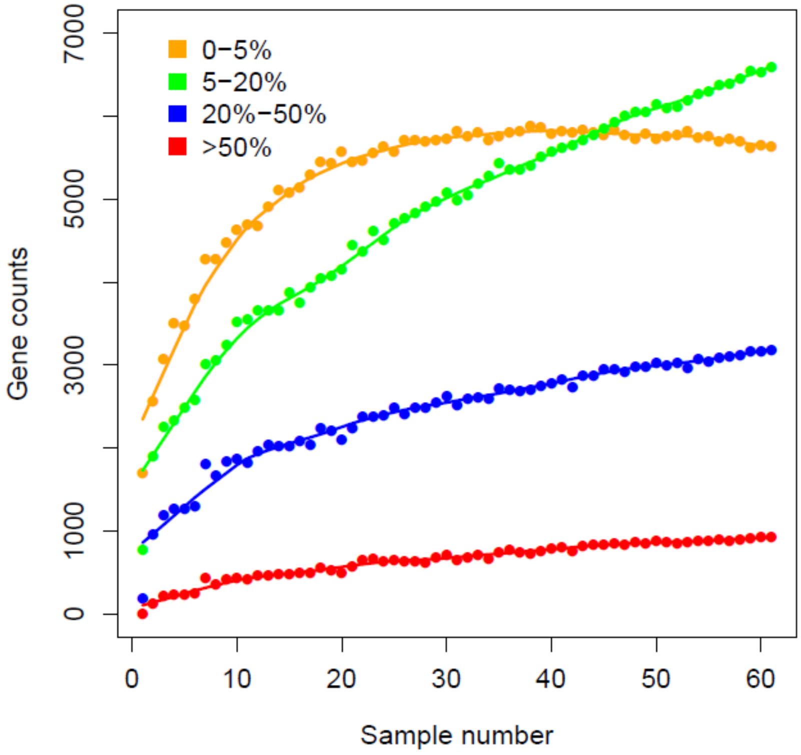

Supplementary Figure 6. Proteomic validation of GENCODE transcripts using the TUM_NCI_60 dataset. In the GENCODE database, transcripts are classified using two schemes. The first classifies transcripts into known, novel, or putative classes, reflecting their similarity to pre-existing models in EntrezGene or Swissprot/Uniprot. The second classifies transcripts into three levels (1-3), which correspond to validated, manually annotated, and automated annotated transcripts, respectively. **(a)** Numbers of peptides mapped to the known, novel and putative transcripts. **(b)** Numbers and percentages of known, novel, and putative transcripts with peptide evidence (column '#Transcript with peptide evidence'), and the numbers and percentages of protein groups in each category (column '#Protein group'). **(c)** Numbers of peptides mapped to the level 1, 2, and 3 transcripts. **(d)** Numbers and percentages of levels 1, 2 and 3 transcripts with peptide evidence (column '#Transcript with peptide evidence'), and the numbers and percentages of protein groups of each level (column '#Protein group'). All results were generated at PSM FDR 0.5%. By overlapping the genome-mapped peptides with gene structures based on range infrastructure, the *proBAM* based approach simplifies the process of using proteomic data to validate genes that are expressed on the protein level. Integrating proteomic data from different samples allows new uses of proteomic data and further becomes a useful resource in the process of genome annotation.

a

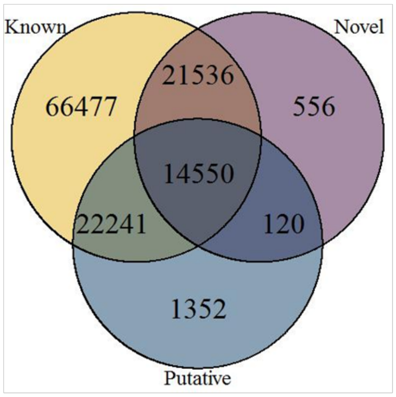

b

	#Total transcript	#Transcript with peptide evidence	#Protein group
KNOWN	67218	46011 (68.45%)	10742 (15.98%)
NOVEL	10218	508 (4.97%)	272 (2.66%)
PUTATIVE	17943	1101 (6.14%)	617 (3.44%)

c

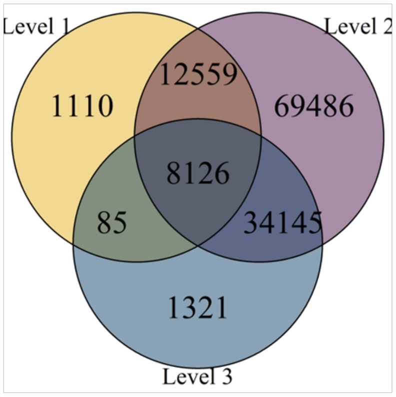

d

	#Total transcript	#Transcript with peptide evidence	#Protein group
Level 1	6505	4073 (62.61%)	850 (13.07%)
Level 2	78288	45277 (57.83%)	10371 (13.25%)
Level 3	10586	651 (6.15%)	410 (3.87%)

Supplementary Figure 7. IGV snapshot of a novel coding region predicted by RNA-Seq data (middle panel) and verified by proteomics data from four cell lines as indicated by different colors (top panel). GENCODE gene annotations are presented in the bottom panel.

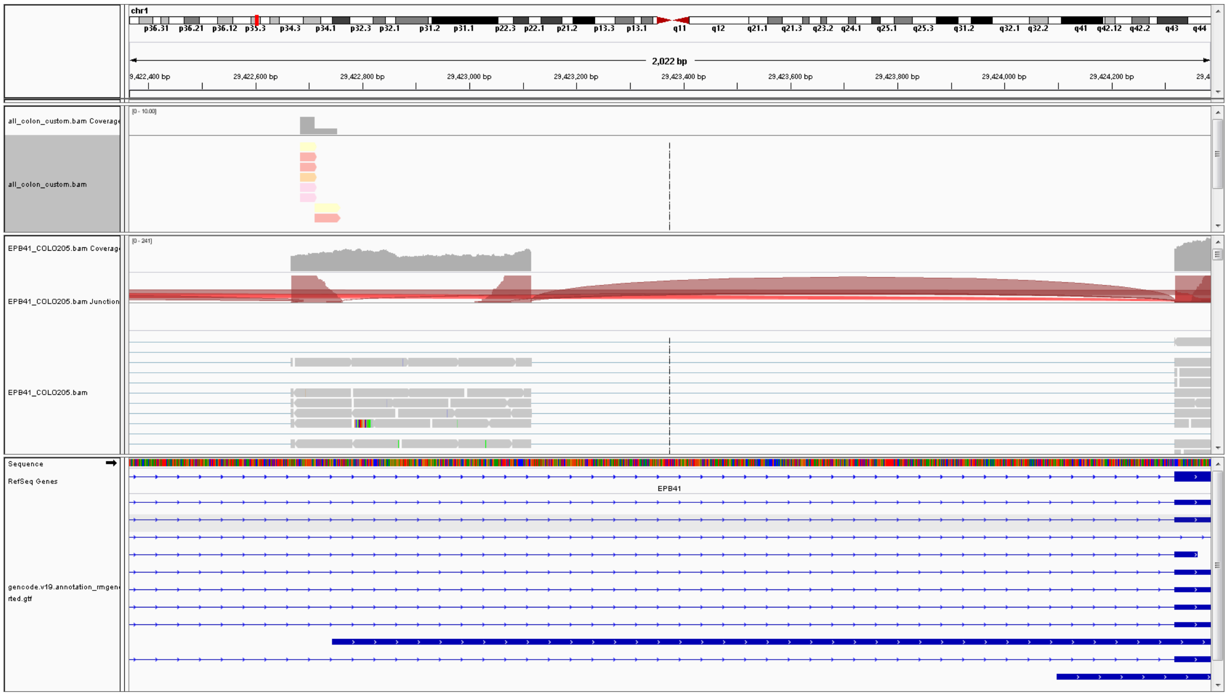

Supplementary Table 1. Definition of the *proBAM* format.

Mandatory fields in BAM and *proBAM*				
**No.**	**NAME**	**TYPE**	**BAM Description**	***proBAM* Description**
**1**	QNAME	String	Query template NAME	Spectrum name
**2**	FLAG	Int	Bitwise FLAG	Bitwise FLAG
**3**	RNAME	String	Reference sequence NAME	Reference sequence NAME
**4**	POS	Int	1-based leftmost mapping POSition	1-based leftmost mapping POSition
**5**	MAPQ	Int	MAPping Quality (Phred-scaled)	255
**6**	CIGAR	String	Extended CIGAR string (operations: MIDNSHP)	CIGAR string
**7**	RNEXT	String	Mate Reference NAME ('=' if same as RNAME)	*
**8**	PNEXT	Int	1-Based leftmost Mate POSition	0
**9**	TLEN	Int	observed Template LENgth	0
**10**	SEQ	String	segment SEQuence	Coding sequence
**11**	QUAL	String	Query QUALity (ASCII-33=Phred base quality)	*

Mandatory fields in *proBAM**		
**TAG**	**TYPE**	**Description**
**NH**	i	Number of genomic locations the peptide mapping to
**XL**	i	Number of peptides the spectrum mapping to
**XP**	Z	Peptide sequence
**XR**	Z	Reference peptide sequence
**XS**	f	PSM score
**XQ**	f	PSM Q-value
**XC**	i	Peptide Charge
**XA**	Z	Whether the peptide is well annotated (0: yes ; 1: partially unknown; 2: totally unknown)
**XM**	Z	Modification
**XN**	i	Number of mis-cleavage
**XT**	i	0: non-tryptic; 1: semi-tryptic; 2: tryptic
**XG**	Z	Peptide type. N: normal peptide; V: variant peptide; J: novel junction peptides; D: decoy; U: unmapped

FLAG description in *proBAM***		
**Bit**	**Description**	**FLAG**
0x00	Peptide map to forward strand	0
0x10	Peptide map to reverse strand	16
0x100	Peptide is NOT the rank=1 peptide for the spectrum	256
0x400	Decoy peptide	1024
0x4	Unmapped peptide	4

* Taking advantage of the flexible optional fields in BAM, we introduced new mandatory fields in *proBAM* for keeping proteomics-specific information. These fields follow the rule TAG:TYPE:VALUE defined by the BAM format. There are three type of VALUE format: i, Singed 32-bit integer; Z, Printable string; f, Single-precision floating number.

** For example, if a peptide maps to the reverse strand and it is not the rank=1 peptide for the spectrum, the FLAG value would be 272 (0x10+0x100)

Supplementary Table 2. Detailed proteomics identification data from the TUM_NCI_60 dataset.

	GENCODE (search)	RefSeq -> GENCODE	GENCODE -> RefSeq	RefSeq (search)
Spectra-peptide-location match	4535568	4444240	4426805	4477872
Spectra	3410633	3385857	3373025	3412732
PSM	3456743	3430709	3418131	3457807
Distinct peptides	141834	138927	137126	139976
Distinct peptide sequence	113534	110369	109172	111219
Peptide mapped to unique genomic location	106852	104809	103610	105529
Within exon peptides	79288	78420	77534	78917
Exon-exon junction peptides	27564	26389	26076	26612
Spanning 2 exon	26851	25727	25454	25937
Spanning 3 exon	662	614	577	625
Peptide mapped to 2 genomic location	4724	3831	3844	3927
Peptide mapped to 3 genomic location	1114	964	983	989
Peptide mapped to 4~10 genomic location	784	709	678	712
Peptide mapped to >10 genomic location	60	59	57	62

Supplementary Table 3. The numbers of identified gene and protein group of four colorectal cancer cell lines in the TUM_NCI_60 and VU_CRC_10 datasets separately or in combination.

Cell Line	TUM_NCI_60 ID	VU_CRC_10	TUM_NCI_60	Combined
**Gene groups**				
COLO205	P001892	4008 (3923)	2486 (2427)	4802 (4725)
HCT116	P003205	3980 (3912)	743 (712)	4251 (4185)
HCT15	P003208	4468 (4382)	2077 (2030)	4972 (4884)
HT29	P001565	4139 (4055)	2591 (2526)	4906 (4819)
**Protein groups**				
COLO205	P001892	4016 (1541)	2487 (962)	4829 (2038)
HCT116	P003205	3992 (1538)	740 (272)	4269 (1691)
HCT15	P003208	4474 (1717)	2087 (779)	4933 (2054)
HT29	P001565	4154 (1601)	2608 (1081)	4939 (2088)

The figure in parentheses indicates the number of protein or gene groups with unique protein or gene ID.

Supplementary Table 4. The genome CDS coverage of four colorectal cancer cell lines in the TUM_NCI_60 and VU_CRC_10 datasets separately or in combination.

Cell Line	TUM_NCI_60 ID	VU_CRC_10	TUM_NCI_60	Combined
COLO205	P001892	0.0247	0.0236	0.0400
HCT116	P003205	0.0268	0.0087	0.0317
HCT15	P003208	0.0293	0.0200	0.0403
HT29	P001565	0.0283	0.0278	0.0449

Supplementary Table 5. The numbers of distinct peptides identified from four colorectal cancer cell lines in the TUM_NCI_60 and VU_CRC_10 datasets separately or in combination.

Cell Line	TUM_NCI_60 ID	VU_CRC_10	TUM_NCI_60	Combined
COLO205	P001892	25427	23570	42983
HCT116	P003205	27154	7866	32416
HCT15	P003208	30378	20002	43416
HT29	P001565	27998	26606	46824
