# Supplementary material for: proBAMsuite, a Bioinformatics Framework for Genome-Based Representation and Analysis of Proteomics Data
Source: Mol Cell Proteomics. 2015 Dec 11;15(3):1164–75. doi: 10.1074/mcp.M115.052860 (PMC4813696; doi:10.1074/mcp.M115.052860)
Supplement: Supplemental Data [file 10.1074_M115.052860_mcp.M115.052860-2.pdf]

A computational pipeline to generate and analyze proBAM files

September 28, 2015

Contents

1 Introduction **1**

2 Building proBAM files using R package *proBAMr* **2**
- 2.1 Preparing annotation files 2
- 2.2 Preparing PSMs table 3
- 2.3 Generate SAM file using `PSMtab2SAM` 4
- 2.4 Convert SAM file to BAM and index 5
- 2.5 Visulize proteomics data in IGV 6

3 Analyzing proBAM files using R package *proBAMtools* **6**
- 3.1 Proteomics data interpretation 7
  - 3.1.1 Summarize proteomics identifications from a proBAM file 7
  - 3.1.2 Sequence coverage analysis of proteomics data 10
- 3.2 Proteomics data assembly 10
  - 3.2.1 Parsimony 10
  - 3.2.2 Generate spectra count table for proteomics data 13
- 3.3 Proteomics data integration 14
  - 3.3.1 Switch annotation 14
  - 3.3.2 Multiple data sets integration 17

4 Session Information **17**

1 Introduction

Recent advances of sequencing technologies have reformed our conception of genomic data analysis, storage and interpretation, instigating more research interest in exploring human proteome at a parallel scale. Shotgun proteomics holds this promise by surveying proteome both qualitatively and quantitatively. Over the last years large amount of proteomics data has been accumulated, an emerging demand is to combine these efforts to catalogue the wide dynamic range of protein expression and complexity of alternative isoforms. However, this task is daunting due to the fact that different studies use varying databases, search engines and assembly tools. Such a challenge calls for an efficient approach of integrating data from different proteomics studies and even with genomic data.

Here we provide a computational pipeline, that maps identified PSMs to the genome in BAM format, a binary format for efficient data storage and fast access in genomic research field. This method differs from other approaches because of its ability of building connections between peptide and genomic location and simultaneously maintaining spectra count information. PSMs are aligned under the same coordination framework regardless of the annotation systems (e.g. RefSeq, ENSEMBL) of the input proteomics data, which enables flexible protein assembly switch between different annotation or at different level (gene or protein). When genomic/transcriptomic information of the same individual is available, this approach allows the co-analysis with -omics data together. In Figure 1, we illustrated the pipeline for generating and analyzing proBAM files. This document provides a step by step tutorial using examples.

Figure 1: A computational pipeline to generate and analyze proBAM files

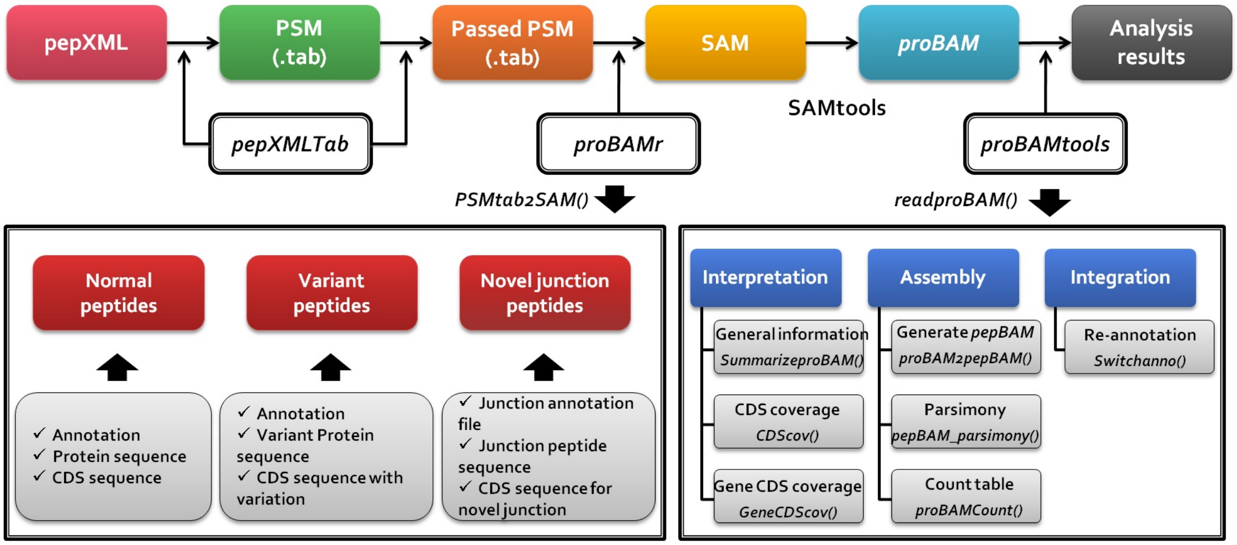

2 Building proBAM files using R package *proBAMr*

2.1 Preparing annotation files

To map proteomics data to the genome, numerous pieces of genome annotation information are needed, such as genome elements region boundary, protein coding sequence and protein sequence et al. It is possible to manually download these data from different public resources (e.g. NCBI, UCSC and ENSEMBL) and then parse them to an appropriate format. To make this process more efficient and autonomous, we provide functions to prepare the gene/transcript annotation files from UCSC, ENSEMBL and GENCODE. The `PrepareAnnotationRefseq` and `PrepareAnnotationEnsembl` were included in another R package *customProDB* `http://bioconductor.org/packages/3.0/bioc/html/customProDB.html`. Here, we provide the function `PrepareAnnotationGENCODE` to prepare the annotation from GENCODE. This function requires users to download GTF file, coding sequence and protein sequence FASTA files from GENCODE ftp `ftp://ftp.sanger.ac.uk/pub/`

gencode/Gencode_human/. Users should use the same version of annotations through the same project annalysis. All the annotations are saved to a specified directory for latter use.

```
> library(proBAMr)

> gtfFile <- system.file("extdata", "test.gtf", package="proBAMr")
> CDSfasta <- system.file("extdata", "coding_seq.fasta", package="proBAMr")
> pepfasta <- system.file("extdata", "pro_seq.fasta", package="proBAMr")
> annotation_path <- tempdir()
> PrepareAnnotationGENCODE(gtfFile, CDSfasta, pepfasta,
+                 annotation_path, dbsnp=NULL,
+                 splice_matrix=FALSE, COSMIC=FALSE)
```

2.2 Preparing PSMs table

After preparing all the annotation files, the R package *pepXMLTab* is used to extract confident PSMs and related information from pepXML files. Other tools are also applicable at this step, as long as it generates similar tabular files, as shown below.

```
> passedPSM <- read.table(system.file("extdata", "passedPSM.tab",
+     package="proBAMr"), sep='\t', header=TRUE)
> passedPSM[1:3, ]

                                   spectrum
1 00463_H12_P003361_B00L_A00_R1.9484.9484.2
2 00463_H12_P003361_B00L_A00_R1.9501.9501.2
3 00463_H12_P003361_B00L_A00_R1.9526.9526.2
                             spectrumNativeID start_scan end_scan
1 controllerType=0 controllerNumber=1 scan=9484       9484     9484
2 controllerType=0 controllerNumber=1 scan=9501       9501     9501
3 controllerType=0 controllerNumber=1 scan=9526       9526     9526
  precursor_neutral_mass assumed_charge index retention_time_sec
1              1945.011              2  1604           5941.112
2              1945.019              2  1614           5951.951
3              1945.016              2  1631           5963.760
  hit_rank            peptide peptide_prev_aa peptide_next_aa
1        1 VNPTVFFDIAVDGEPLGR               M               V
2        1 VNPTVFFDIAVDGEPLGR               M               V
3        1 VNPTVFFDIAVDGEPLGR               M               V
                                                                                                     protei
1 ENST00000415933.1|ENSG00000196262.9|OTTHUMG00000023687.5|OTTHUMT00000341788.1|PPIA-007|PPIA|12
2 ENST00000415933.1|ENSG00000196262.9|OTTHUMG00000023687.5|OTTHUMT00000341788.1|PPIA-007|PPIA|12
3 ENST00000415933.1|ENSG00000196262.9|OTTHUMG00000023687.5|OTTHUMT00000341788.1|PPIA-007|PPIA|12
  num_tot_proteins calc_neutral_pep_mass    massdiff num_tol_term
1                4              1944.995 -0.01667663            2
2                4              1944.995 -0.02461120            2
```

```
3                4             1944.995 -0.02192565           2
  num_missed_cleavages num_matched_ions tot_num_ions      mvh
1                    0               21           31 46.20442
2                    0               26           31 60.50605
3                    0               22           31 52.51659
  mzFidelity    xcorr modification NTT
1   81.97849 4.276119         <NA>   1
2  104.25397 5.334539         <NA>   1
3   86.18693 5.057394         <NA>   1
```

2.3 Generate SAM file using PSMtab2SAM

The function PSMtab2SAM first finds the peptide location in protein sequences, then maps the coding sequence of the peptide back to the genome according to the annotation.

```
> load(system.file("extdata/GENCODE", "exon_anno.RData", package="proBAMr"))
> load(system.file("extdata/GENCODE", "proseq.RData", package="proBAMr"))
> load(system.file("extdata/GENCODE", "procodingseq.RData", package="proBAMr"))
> options(stringsAsFactors=FALSE)
> passedPSM <- read.table(system.file("extdata", "passedPSM.tab",
+     package="proBAMr"), sep='\t', header=TRUE)
> outfile <- paste(tempdir(), '/test.sam', sep='')
> PSMtab2SAM(passedPSM, XScolumn='mvh', exon, proteinseq,
+     procodingseq, outfile)
> SAM <- read.table(file=outfile, sep='\t')
> dim(SAM)
```

```
[1] 40 21
```

```
> SAM[20:27, ]
```

```
                                       V1 V2    V3        V4  V5
20 00463_H12_P003361_B00L_A00_R1.0.1.7307 16 chr11  65622810 255
21 00463_H12_P003361_B00L_A00_R1.0.1.7350  0  chr7  44839340 255
22 00463_H12_P003361_B00L_A00_R1.0.1.7441  0  chr7  44836381 255
23 00463_H12_P003361_B00L_A00_R1.0.1.7457  0  chr7  44836381 255
24 00463_H12_P003361_B00L_A00_R1.0.1.7898 16  chr5 133509648 255
25 00463_H12_P003361_B00L_A00_R1.0.1.7915 16  chr5 133509648 255
26 00463_H12_P003361_B00L_A00_R1.0.1.7933 16  chr5 133509648 255
27 00463_H12_P003361_B00L_A00_R1.0.1.7952 16  chr1  26230237 255
            V6 V7 V8 V9
20         42M  *  0  0
21         45M  *  0  0
22 12M2453N24M  *  0  0
23 12M2453N24M  *  0  0
24         51M  *  0  0
```

```
25         51M  *  0  0
26         51M  *  0  0
27         39M  *  0  0
                                                      V10 V11    V12
20          CAAAGGCTTGCCCTCCAGGGAGATGACGGCACTGCCCCCCAG   * XA:Z:0
21       TCCATCTATGGGGAGAAATTTGAAGATGAGAACTTCATCCTAAAG   * XA:Z:0
22                GTCTCCTTTGAGCTGTTTGCAGACAAGGTCCCAAAG   * XA:Z:0
23                GTCTCCTTTGAGCTGTTTGCAGACAAGGTCCCAAAG   * XA:Z:0
24 TTTGGCAATTTCCACATCAACTTCAAATATCTCTCCATCAGAACTCTGCAA   * XA:Z:0
25 TTTGGCAATTTCCACATCAACTTCAAATATCTCTCCATCAGAACTCTGCAA   * XA:Z:0
26 TTTGGCAATTTCCACATCAACTTCAAATATCTCTCCATCAGAACTCTGCAA   * XA:Z:0
27             CCGAGGGCTGAGAATCAGCTCAAAAGCCTGGCCTGAGGC   * XA:Z:0
      V13    V14                    V15    V16          V17    V18
20 NH:i:1 XL:i:1    XP:Z:LGGSAVISLEGKPL XC:i:2 XS:f:30.6722 XM:Z:-
21 NH:i:1 XL:i:1   XP:Z:SIYGEKFEDENFILK XC:i:2 XS:f:22.4909 XM:Z:-
22 NH:i:1 XL:i:1      XP:Z:VSFELFADKVPK XC:i:2  XS:f:21.321 XM:Z:-
23 NH:i:1 XL:i:1      XP:Z:VSFELFADKVPK XC:i:2 XS:f:25.2581 XM:Z:-
24 NH:i:1 XL:i:1 XP:Z:LQSSDGEIFEVDVEIAK XC:i:2 XS:f:30.9829 XM:Z:-
25 NH:i:1 XL:i:1 XP:Z:LQSSDGEIFEVDVEIAK XC:i:2 XS:f:42.8235 XM:Z:-
26 NH:i:1 XL:i:1 XP:Z:LQSSDGEIFEVDVEIAK XC:i:2 XS:f:33.9951 XM:Z:-
27 NH:i:1 XL:i:1     XP:Z:ASGQAFELILSPR XC:i:2 XS:f:23.4655 XM:Z:-
      V19    V20    V21
20 XN:i:0 XT:i:2 XG:Z:N
21 XN:i:1 XT:i:2 XG:Z:N
22 XN:i:1 XT:i:2 XG:Z:N
23 XN:i:1 XT:i:2 XG:Z:N
24 XN:i:0 XT:i:2 XG:Z:N
25 XN:i:0 XT:i:2 XG:Z:N
26 XN:i:0 XT:i:2 XG:Z:N
27 XN:i:0 XT:i:2 XG:Z:N
```

2.4 Convert SAM file to BAM and index

Add the header to the SAM file. Converted them to the binary BAM files using samtools `http://samtools.sourceforge.net/`. Sort and index them for fast access.

The bullet list below summarizes the steps after the SAM file been generated.

```
> paste('cat header test.sam > test_header.sam')

[1] "cat header test.sam > test_header.sam"

> paste('samtools view -S -b test_header.sam > test_header.bam')

[1] "samtools view -S -b test_header.sam > test_header.bam"

> paste('samtools sort test_header.bam > test_header_sort')
```

```
[1] "samtools sort test_header.bam > test_header_sort"

> paste('samtools index test_header_sort')

[1] "samtools index test_header_sort"
```

2.5 Visulize proteomics data in IGV

The proBAM files can be visulized in IGV directly. Furthermore, users can co-visulize their proteomics data with the paired genomics/transcriptomics data, as shown in Fig 2.

Figure 2: IGV snapshot of a homozygous mutation in gene ALDH1B1 in both proteomics and RNA-Seq data (inside read box)

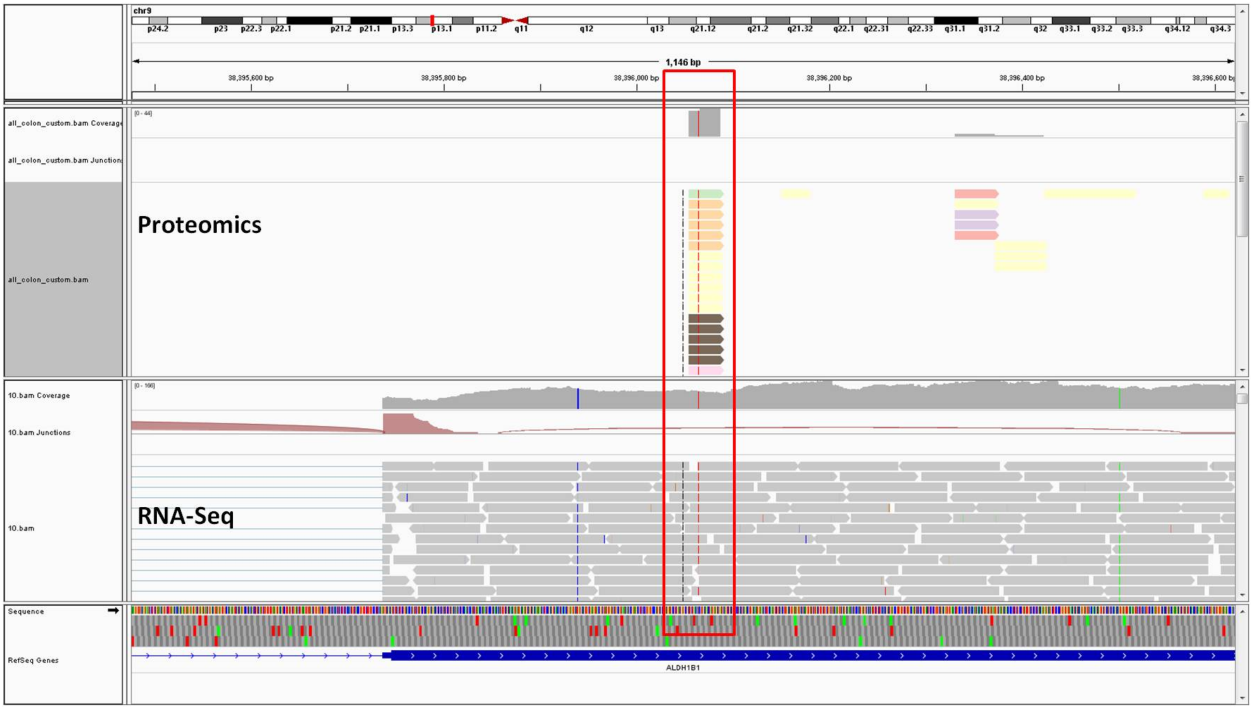

3 Analyzing proBAM files using R package *proBAMtools*

This R package, *proBAMtools*, is designed to perform various analyses based on the proBAM files, including tools for proteomics data interpretation, assembly, and integration. This document provides a step by step tutorial using a toy genome, which only contains two genes.

3.1 Proteomics data interpretation

3.1.1 Summarize proteomics identifications from a proBAM file

The function `SummarizeproBAM` is used to summarize the proteomics identifications from a proBAM file. In addition to the number of identifiable spectra, PSMs and unique peptide, it is easy to identify genomic regions from which the peptides are derived with the genomic sequence mapping information available in the proBAM files. The function calculates the number of peptides mapped to unique or multiple genomic locations. And for peptides mapped to unique genomic locations, the function also provides the number of within exon peptides and exon-exon junction peptides.

```
> library(proBAMtools)
```

```
> proBAMFile <- system.file("extdata", "test.bam", package="proBAMtools")
> glan_proBAM <- readproBAM(proBAMFile)
> glan_proBAM
```

```
GAlignments object with 146 alignments and 15 metadata columns:
        seqnames strand       cigar    qwidth     start       end
           <Rle>  <Rle> <character> <integer> <integer> <integer>
    [1]    chr12      +         27M        27  25357166  25357192
    [2]    chr12      +         27M        27  25357166  25357192
    [3]    chr12      +         27M        27  25357166  25357192
    [4]    chr12      +         27M        27  25357166  25357192
    [5]    chr12      +         27M        27  25357166  25357192
    ...      ...    ...         ...       ...       ...       ...
  [142]    chr12      - 12M17861N18M       30  25380335  25398225
  [143]    chr12      - 12M17861N18M       30  25380335  25398225
  [144]    chr12      -         54M        54  25398262  25398315
  [145]    chr12      -         33M        33  25398271  25398303
  [146]    chr12      -         33M        33  25398271  25398303
            width     njunc   |
        <integer> <integer>   |
    [1]        27         0   |
    [2]        27         0   |
    [3]        27         0   |
    [4]        27         0   |
    [5]        27         0   |
    ...       ...       ... ...
  [142]     17891         1   |
  [143]     17891         1   |
  [144]        54         0   |
  [145]        33         0   |
  [146]        33         0   |
                                                    qname      flag
                                              <character> <integer>
    [1]           klc_100908x_PH_P10_DLD_1_A12.0.1.8807          0
```

```
  [2]            mam_072308x_PH_P8_HCT_116_B12.0.1.7396         0
  [3] klc_070108x_PH_P7_HCT_15_C12_080712110959.0.1.7206         0
  [4]              klc_080408x_PH_P8_HT_29_E12.0.1.7619         0
  [5]            klc_080408x_PH_P8_LS174_T_F12.0.1.7883         0
  ...                                                ...       ...
[142]             klc_090308x_PH_P7a_SW480_J05.0.1.3992        16
[143]             klc_090308x_PH_P7a_SW480_J05.0.1.4008        16
[144]             mam_072308x_PH_P8_HCT_15_C11.0.1.6201        16
[145]           klc_082108x_PH_P7a_CaCo2_G15_1.0.1.4080        16
[146]             klc_090308x_PH_P7a_SW480_J15.0.1.4920        16
                                                     seq
                                          <DNAStringSet>
  [1]                           GAGCTAGAAGCTTTGTACTTCCTTAGG
  [2]                           GAGCTAGAAGCTTTGTACTTCCTTAGG
  [3]                           GAGCTAGAAGCTTTGTACTTCCTTAGG
  [4]                           GAGCTAGAAGCTTTGTACTTCCTTAGG
  [5]                           GAGCTAGAAGCTTTGTACTTCCTTAGG
  ...                                                   ...
[142]                        CCTGTAGGAATCCTCTATTGTTGGATCATA
[143]                        CCTGTAGGAATCCTCTATTGTTGGATCATA
[144] CAAGGCACTCTTGCCTACGTCACCAGCTCCAACTACCACAAGTTTATATTCAGT
[145]                     CTTGCCTACGCCACCAGCTCCAACTACCACAAG
[146]                     CTTGCCTACGCCAACAGCTCCAACTACCACAAG
             NH        XL                  XP                  XR
      <integer> <integer>         <character>         <character>
  [1]         1         1           ELEALYFLR           ELEALYFLR
  [2]         1         1           ELEALYFLR           ELEALYFLR
  [3]         1         1           ELEALYFLR           ELEALYFLR
  [4]         1         1           ELEALYFLR           ELEALYFLR
  [5]         1         1           ELEALYFLR           ELEALYFLR
  ...       ...       ...                 ...                 ...
[142]         6         1          YDPTIEDSYR          YDPTIEDSYR
[143]         6         1          YDPTIEDSYR          YDPTIEDSYR
[144]         1         1 TEYKLVVVGAGDVGKSAL TEYKLVVVGAGGVGKSAL
[145]         4         1         LVVVGAGGVGK         LVVVGAGGVGK
[146]         1         1         LVVVGAVGVGK         LVVVGAGGVGK
             XS        XQ        XC          XA          XM
      <numeric> <logical> <integer> <character> <character>
  [1]   39.1477      <NA>         2           0          NA
  [2]   39.2369      <NA>         2           0          NA
  [3]   45.3205      <NA>         2           0          NA
  [4]   50.0433      <NA>         2           0          NA
  [5]   52.9704      <NA>         2           0          NA
  ...       ...       ...       ...         ...         ...
[142]   49.2137      <NA>         2           0          NA
```

```
[143]   32.8392      <NA>         2           0          NA
[144]   41.6893      <NA>         4           0          NA
[145]   49.9949      <NA>         2           0          NA
[146]   45.6955      <NA>         2           0          NA
               XN         XT          XG
      <integer> <integer> <character>
  [1]         0          2           N
  [2]         0          2           N
  [3]         0          2           N
  [4]         0          2           N
  [5]         0          2           N
  ...       ...        ...         ...
[142]         0          2           N
[143]         0          2           N
[144]         2          0           V
[145]         0          2           N
[146]         0          2           V
-------
seqinfo: 217 sequences from an unspecified genome
```

> SummarizeproBAM(glan_proBAM)

```
                               Spectra
                                   146
                                   PSM
                                   146
                      Distinct_peptide
                                    14
                  Distinct_peptide_seq
                                    12
Peptide map to unique genomic location
                                    12
                  Peptides within exon
                                     9
               Peptides spanning exons
                                     3
             Peptides spanning 2 exons
                                     3
             Peptides spanning 3 exons
                                    NA
           Peptides spanning > 3 exons
                                     0
     Peptide map to 2 genomic location
                                    NA
     Peptide map to 3 genomic location
                                    NA
```

```
  Peptide map to 4-10 genomic location
                                    NA
   Peptide map to >10 genomic location
                                    NA
```

3.1.2 Sequence coverage analysis of proteomics data

While existing proteomics data formats only report sequence coverage for individual proteins, the proBAM format contains genome information, thereby allowing *proBAMtools* to provide sequence coverage reports at the genome, chromosome, and individual gene levels. The function `CDScov` is used to calculate sequence coverage at the whole genome or chromosome level.

```
> txdb <- loadDb(system.file("extdata/anno_ensembl", "txdb.sqlite",
+     package="proBAMtools"))
> CDScov(glan_proBAM, txdb, bychr=FALSE)

[1] 0.1579892

> CDScov(glan_proBAM, txdb, bychr=TRUE)

     chr1      chr2      chr3      chr4      chr5      chr6      chr7 
      NaN       NaN       NaN       NaN       NaN       NaN       NaN 
     chr8      chr9     chr10     chr11     chr12     chr13     chr14 
      NaN       NaN       NaN       NaN 0.1579892       NaN       NaN 
    chr15     chr16     chr17     chr18     chr19     chr20     chr21 
      NaN       NaN       NaN       NaN       NaN       NaN       NaN 
    chr22      chrX      chrY 
      NaN       NaN       NaN 
```

And function `GeneCDScov` is provided to calculate the sequence coverage for individual genes.

```
> GeneCDScov(glan_proBAM, txdb)

ENSG00000133703 ENSG00000205707 
     0.33474576      0.02288136 
```

3.2 Proteomics data assembly

3.2.1 Parsimony

In a typical proteomics study, parsimony analysis is usually used to derive a minimal protein list that is sufficient to explain the observed peptide identification. We provide a function `proBAM2pepBAM` to convert a PSMs based proBAM file to the peptide based proBAM file.

```
> pepBAM <- proBAM2pepBAM(glan_proBAM)
> pepBAM
```

```
GAlignments object with 16 alignments and 13 metadata columns:
       seqnames strand        cigar    qwidth     start       end
          <Rle>  <Rle>  <character> <integer> <integer> <integer>
   [1]    chr12      +          27M        27  25357166  25357192
   [2]    chr12      -  33M15702N3M        36  25362813  25378550
   [3]    chr12      -  33M15702N3M        36  25362813  25378550
   [4]    chr12      -          30M        30  25368462  25368491
   [5]    chr12      -          30M        30  25368462  25368491
   ...      ...    ...          ...       ...       ...       ...
  [12]    chr12      - 12M17861N18M        30  25380335  25398225
  [13]    chr12      - 12M17861N18M        30  25380335  25398225
  [14]    chr12      -          54M        54  25398262  25398315
  [15]    chr12      -          33M        33  25398271  25398303
  [16]    chr12      -          33M        33  25398271  25398303
            width     njunc   |    qwidth     njunc      flag
        <integer> <integer>   | <integer> <integer> <integer>
   [1]         27         0   |        27         0         0
   [2]      15738         1   |        36         1        16
   [3]      15738         1   |        36         1        16
   [4]         30         0   |        30         0        16
   [5]         30         0   |        30         0        16
   ...        ...       ... ...       ...       ...       ...
  [12]      17891         1   |        30         1        16
  [13]      17891         1   |        30         1        16
  [14]         54         0   |        54         0        16
  [15]         33         0   |        33         0        16
  [16]         33         0   |        33         0        16
                                                          seq
                                                  <character>
   [1]                            GAGCTAGAAGCTTTGTACTTCCTTAGG
   [2]                   TCGAACTAATGTATAGAAGGCATCATCAACACCCTG
   [3]                   TCGAACTAATGTATAGAAGGCATCATCAACACCCTG
   [4]                         CCTCACCAATGTATAAAAAGCATCCTCCAC
   [5]                         CCTCACCAATGTATAAAAAGCATCCTCCAC
   ...                                                    ...
  [12]                         CCTGTAGGAATCCTCTATTGTTGGATCATA
  [13]                         CCTGTAGGAATCCTCTATTGTTGGATCATA
  [14] CAAGGCACTCTTGCCTACGTCACCAGCTCCAACTACCACAAGTTTATATTCAGT
  [15]                      CTTGCCTACGCCACCAGCTCCAACTACCACAAG
  [16]                      CTTGCCTACGCCAACAGCTCCAACTACCACAAG
                XA        NH                XP                XR
       <character> <integer>       <character>       <character>
   [1]           0         1         ELEALYFLR         ELEALYFLR
   [2]           0         1      QGVDDAFYTLVR      QGVDDAFYTLVR
   [3]           0         1      QGVDDAFYTLVR      QGVDDAFYTLVR
```

```
  [4]           0         4         VEDAFYTLVR         VEDAFYTLVR
  [5]           0         4         VEDAFYTLVR         VEDAFYTLVR
  ...         ...       ...                ...                ...
 [12]           0         6         YDPTIEDSYR         YDPTIEDSYR
 [13]           0         6         YDPTIEDSYR         YDPTIEDSYR
 [14]           0         1 TEYKLVVVGAGDVGKSAL TEYKLVVVGAGGVGKSAL
 [15]           0         4        LVVVGAGGVGK        LVVVGAGGVGK
 [16]           0         1        LVVVGAVGVGK        LVVVGAGGVGK
             XC               XM        XN        XT          XG
      <integer>      <character> <integer> <integer> <character>
  [1]         2                -         0         2           N
  [2]         2                -         0         2           N
  [3]         2 1;111.0320285114         0         2           N
  [4]         2                -         0         1           N
  [5]         2                -         0         2           N
  ...       ...              ...       ...       ...         ...
 [12]         2                -         0         2           N
 [13]         2                -         0         1           N
 [14]         4                -         2         0           V
 [15]         2                -         0         2           N
 [16]         2                -         0         2           V
 -------
 seqinfo: 1 sequence from an unspecified genome; no seqlengths
```

In function `pepBAM_parsimony`, a previously published parsimony procedure is usde to generate a minimal list of identified proteins or genes. The procedure relies on bipartite graph modeling. What distinguishes the proBAM approach from the previous approach is the utilization of genomic location information in the construction of the bipartite graph. Specifically, the proBAM-based approach can relate peptides to different types of genomic elements such as exons, transcript isoforms, or genes based on their overlapping relationship on the genome. Consequently, inference can be made at both protein level and gene level. Moreover, inference can be made based on different versions of genome annotations.

```
> #gene level parsimony
> cdsByGe <- cdsBy(txdb, "gene", use.names=FALSE)
> gegp <- pepBAM_parsimony(pepBAM, cdsByGe)
> gegp

     Group
[1,] "ENSG00000205707"
[2,] "ENSG00000133703"
     Peptides
[1,] "ELEALYFLR 2 -_pep"
[2,] "QGVDDAFYTLVR 2 -_pep:QGVDDAFYTLVR 2 1;111.0320285114_pep:VEDAFYTLVR 2 -_pep:QAQDLAR 1 1;11
     #inGroup #ofPeptide
```

```
[1,] "1"      "1"
[2,] "1"      "13"
```

```
> #protein level parsimony
> cdsByTx <- cdsBy(txdb, "tx", use.names=TRUE)
> progp <- pepBAM_parsimony(pepBAM, cdsByTx)
> progp
```

```
     Group
[1,] "ENST00000381356:ENST00000556351:ENST00000556885:ENST00000556927:ENST00000557540"
[2,] "ENST00000311936"
[3,] "ENST00000256078"
     Peptides
[1,] "ELEALYFLR 2 -_pep"
[2,] "QGVDDAFYTLVR 2 -_pep:QGVDDAFYTLVR 2 1;111.0320285114_pep:QAQDLAR 1 1;111.0320285114_pep:DS
[3,] "VEDAFYTLVR 2 -_pep:QAQDLAR 1 1;111.0320285114_pep:DSEDVPMVLVGNKCDLPSR 3 7;147.0353996062;1
     #inGroup #ofPeptide
[1,] "5"      "1"
[2,] "1"      "12"
[3,] "1"      "11"
```

3.2.2 Generate spectra count table for proteomics data

proBAMtools generates count tables on the basis of genomic structure of the genes. Both spectral count and peptide count tables are provided at protein isoform- and gene-levels, respectively, and both overall counts and gene-specific or isoform-specific counts are reported. The overall spectral count for a gene or an isoform respectively sums up all PSMs associated with the gene or the isoform, whereas the gene-specific or isoform-specific spectral count, respectively, sums up only PSMs associated specifically with the gene or the isoform. The same conditions also apply to peptide counting. Count data are provided for individual genes and proteins. By integrating these data with the minimal protein or gene group lists resulting from the parsimony analysis, count tables can be generated for the confidently identified genes or proteins.

```
> ##gene level peptide count
> cdsByGe <- cdsBy(txdb, "gene", use.names=FALSE)
> proBAMCount(pepBAM, cdsByGe)
```

```
                specific overall
ENSG00000133703        8      15
ENSG00000205707        1       1
```

```
> ##gene level spectra count
> proBAMCount(glan_proBAM, cdsByGe)
```

```
                specific overall
ENSG00000133703       78     139
ENSG00000205707        7       7
```

```
> ##protein level peptide count
> cdsByTx <- cdsBy(txdb, "tx", use.names=TRUE)
> proBAMCount(pepBAM, cdsByTx)

                specific overall
ENST00000256078        0      13
ENST00000311936        2      13
ENST00000381356        0       1
ENST00000553788        0       0
ENST00000554266        0       0
ENST00000555151        0       0
ENST00000555711        0       0
ENST00000556131        0       3
ENST00000556198        0       0
ENST00000556351        0       1
ENST00000556402        0       0
ENST00000556885        0       1
ENST00000556927        0       1
ENST00000557334        0       3
ENST00000557540        0       1

> ##protein level spectra count
> proBAMCount(glan_proBAM, cdsByTx)

                specific overall
ENST00000256078        0      85
ENST00000311936       54     124
ENST00000381356        0       7
ENST00000553788        0       0
ENST00000554266        0       0
ENST00000555151        0       0
ENST00000555711        0       0
ENST00000556131        0       3
ENST00000556198        0       0
ENST00000556351        0       7
ENST00000556402        0       0
ENST00000556885        0       7
ENST00000556927        0       7
ENST00000557334        0       3
ENST00000557540        0       7
```

3.3 Proteomics data integration

3.3.1 Switch annotation

Using genomic sequence as a common reference, proBAM provides a natural solution for proteomics data integration. PSMs align with the genome regardless of the protein database used in the pro-

teomics study, thereby allowing flexible switching between different gene annotation schemes. We developed a function Switchanno, that use a three-step procedure to switch gene annotation scheme for proBAM files, which includes: 1) Removing peptides with any region out of the CDSs of the new annotation scheme; 2) Removing peptides with any region inconsistent with the gene structure of the new scheme; 3) Removing peptides that are out of frame in the new scheme. Accordingly, proteomics datasets generated by searching against different databases can be converted to the same gene annotation scheme for integration.

```
> new_annotation_path <- system.file("extdata/anno_refseq/",
+     package="proBAMtools")
> Switchanno(glan_proBAM, new_annotation_path)
```

```
GAlignments object with 139 alignments and 15 metadata columns:
        seqnames strand       cigar    qwidth     start       end
           <Rle>  <Rle> <character> <integer> <integer> <integer>
    [1]    chr12      - 33M15702N3M        36  25362813  25378550
    [2]    chr12      - 33M15702N3M        36  25362813  25378550
    [3]    chr12      - 33M15702N3M        36  25362813  25378550
    [4]    chr12      - 33M15702N3M        36  25362813  25378550
    [5]    chr12      - 33M15702N3M        36  25362813  25378550
    ...      ...    ...         ...       ...       ...       ...
  [135]    chr12      -         45M        45  25378647  25378691
  [136]    chr12      -         39M        39  25378647  25378685
  [137]    chr12      -         54M        54  25398262  25398315
  [138]    chr12      -         33M        33  25398271  25398303
  [139]    chr12      -         33M        33  25398271  25398303
            width     njunc   |
        <integer> <integer>   |
    [1]     15738         1   |
    [2]     15738         1   |
    [3]     15738         1   |
    [4]     15738         1   |
    [5]     15738         1   |
    ...       ...       ... ...
  [135]        45         0   |
  [136]        39         0   |
  [137]        54         0   |
  [138]        33         0   |
  [139]        33         0   |
                                            qname      flag
                                      <character> <integer>
    [1]    klc_080408x_PH_P8_CaCo2_G08.0.1.6578        16
    [2]    klc_080408x_PH_P8_CaCo2_G08.0.1.6645        16
    [3]    klc_080408x_PH_P8_CaCo2_G08.0.1.7102        16
    [4]    klc_080408x_PH_P8_CaCo2_G08.0.1.7110        16
    [5] klc_082108x_PH_P7a_CaCo2_G08_1.0.1.6905        16
```

```
  ...                                  ...        ...
[135]   klc_090308x_PH_P7a_SW480_J15.0.1.5465        16
[136]    mam_20081229x_P10_SW480_J03.0.1.5910        16
[137]   mam_072308x_PH_P8_HCT_15_C11.0.1.6201        16
[138] klc_082108x_PH_P7a_CaCo2_G15_1.0.1.4080        16
[139]   klc_090308x_PH_P7a_SW480_J15.0.1.4920        16
                                                       seq
                                            <DNAStringSet>
  [1]                  TCGAACTAATGTATAGAAGGCATCATCAACACCCTG
  [2]                  TCGAACTAATGTATAGAAGGCATCATCAACACCCTG
  [3]                  TCGAACTAATGTATAGAAGGCATCATCAACACCCTG
  [4]                  TCGAACTAATGTATAGAAGGCATCATCAACACCCTG
  [5]                  TCGAACTAATGTATAGAAGGCATCATCAACACCCTG
  ...                                                  ...
[135]         TTTATTTCCTACTAGGACCATAGGTACATCTTCAGAGTCCTTAAC
[136]               TTTATTTCCTACTAGGACCATAGGTACATCTTCAGAGTC
[137] CAAGGCACTCTTGCCTACGTCACCAGCTCCAACTACCACAAGTTTATATTCAGT
[138]                     CTTGCCTACGCCACCAGCTCCAACTACCACAAG
[139]                     CTTGCCTACGCCAACAGCTCCAACTACCACAAG
             NH        XL                 XP                XR
      <integer> <integer>        <character>       <character>
  [1]         1         1       QGVDDAFYTLVR      QGVDDAFYTLVR
  [2]         1         1       QGVDDAFYTLVR      QGVDDAFYTLVR
  [3]         1         1       QGVDDAFYTLVR      QGVDDAFYTLVR
  [4]         1         1       QGVDDAFYTLVR      QGVDDAFYTLVR
  [5]         1         1       QGVDDAFYTLVR      QGVDDAFYTLVR
  ...       ...       ...                ...               ...
[135]         1         1    VKDSEDVPMVLVGNK    VKDSEDVPMVLVGNK
[136]         1         1      DSEDVPMVLVGNK      DSEDVPMVLVGNK
[137]         1         1 TEYKLVVVGAGDVGKSAL TEYKLVVVGAGGVGKSAL
[138]         4         1        LVVVGAGGVGK       LVVVGAGGVGK
[139]         1         1        LVVVGAVGVGK       LVVVGAGGVGK
             XS        XQ        XC          XA               XM
      <numeric> <logical> <integer> <character>      <character>
  [1]   39.3926      <NA>         2           0               NA
  [2]   41.2553      <NA>         2           0               NA
  [3]   52.9468      <NA>         2           0 1;111.0320285114
  [4]   45.3983      <NA>         2           0 1;111.0320285114
  [5]   59.9704      <NA>         2           0               NA
  ...       ...       ...       ...         ...              ...
[135]   48.6780      <NA>         3           0               NA
[136]   57.9800      <NA>         2           0 7;147.0353996062
[137]   41.6893      <NA>         4           0               NA
[138]   49.9949      <NA>         2           0               NA
[139]   45.6955      <NA>         2           0               NA
```

```
              XN        XT          XG
       <integer> <integer> <character>
    [1]         0         2           N
    [2]         0         2           N
    [3]         0         2           N
    [4]         0         2           N
    [5]         0         2           N
    ...       ...       ...         ...
  [135]         1         2           N
  [136]         0         2           N
  [137]         2         0           V
  [138]         0         2           N
  [139]         0         2           V
  -------
  seqinfo: 24 sequences from an unspecified genome
```

```
> length(glan_proBAM)
```

```
[1] 146
```

```
> length(Switchanno(glan_proBAM, new_annotation_path))
```

```
[1] 139
```

3.3.2 Multiple data sets integration

proBAM files are generated from individual studies using *proBAMr* based on user-specified PSM FDRs. Because PSMs in all resulting proBAM files are mapped to the genome and thus aligned in the same coordinate system, they can be easily combined into one proBAM file. All PSMs in this combined proBAM file can be re-annotated according to a user-specified gene annotation scheme as described above.

4 Session Information

```
R version 3.1.1 (2014-07-10)
Platform: x86_64-unknown-linux-gnu (64-bit)

locale:
 [1] LC_CTYPE=en_US.UTF-8       LC_NUMERIC=C
 [3] LC_TIME=en_US.UTF-8        LC_COLLATE=en_US.UTF-8
 [5] LC_MONETARY=en_US.UTF-8    LC_MESSAGES=en_US.UTF-8
 [7] LC_PAPER=en_US.UTF-8       LC_NAME=C
 [9] LC_ADDRESS=C               LC_TELEPHONE=C
[11] LC_MEASUREMENT=en_US.UTF-8 LC_IDENTIFICATION=C

attached base packages:
```

```
[1] stats4    parallel  stats     graphics  grDevices utils
[7] datasets  methods   base

other attached packages:
 [1] proBAMtools_0.99.0      Matrix_1.2-2
 [3] igraph_1.0.1            GenomicFeatures_1.18.7
 [5] GenomicAlignments_1.2.2 Rsamtools_1.18.3
 [7] Biostrings_2.34.1       XVector_0.6.0
 [9] GenomicRanges_1.18.4    proBAMr_1.3.2
[11] AnnotationDbi_1.28.2    GenomeInfoDb_1.2.5
[13] Biobase_2.26.0          IRanges_2.0.1
[15] S4Vectors_0.4.0         BiocGenerics_0.12.1

loaded via a namespace (and not attached):
 [1] base64enc_0.1-3    BatchJobs_1.6      BBmisc_1.9
 [4] BiocParallel_1.0.3 biomaRt_2.22.0     bitops_1.0-6
 [7] brew_1.0-6         checkmate_1.6.2    codetools_0.2-14
[10] DBI_0.3.1          digest_0.6.8       fail_1.2
[13] foreach_1.4.2      grid_3.1.1         iterators_1.0.7
[16] lattice_0.20-33    magrittr_1.5       RCurl_1.95-4.7
[19] RSQLite_1.0.0      rtracklayer_1.26.3 sendmailR_1.2-1
[22] stringi_0.5-5      stringr_1.0.0      tools_3.1.1
[25] XML_3.98-1.3       zlibbioc_1.12.0
```
