# Supplementary material for: proBAMsuite, a Bioinformatics Framework for Genome-Based Representation and Analysis of Proteomics Data
Source: Mol Cell Proteomics. 2015 Dec 11;15(3):1164–75. doi: 10.1074/mcp.M115.052860 (PMC4813696; doi:10.1074/mcp.M115.052860)
Supplement: Supplemental Data [file 10.1074_M115.052860_mcp.M115.052860-3.pdf]

```
> library(Rsamtools)
> library(GenomicFeatures)
> library(GenomicAlignments)
> library(proBAMtools)
> options(stringsAsFactors=FALSE)
> outpath <- '/res/merge/COLO205/'
> ########load GENCODE annotation
> gencode_v19 <- '/annotation/GENCODE/v19/'
> txdb <- loadDb(file=paste(gencode_v19, '/txdb.sqlite', sep=''))
> load(paste(gencode_v19, '/ids.RData', sep=''))
> cdsByTx <- cdsBy(txdb, "tx", use.names=TRUE)
> cdsByGe <- cdsBy(txdb, "gene", use.names=FALSE)
> ##################################
> # COLO205 in VU_CRC10 data set
> ##################################
> ## Size of proBAM file : 2.53MB
> bamFile <- '/VU_CRC10/COLO205.bam'
> #########load proBAM file, takes ~3s
> galn_custom <- readproBAM(bamFile)
> #########summarize proteomics identifications, takes ~9s
> SummarizeproBAM(galn_custom)
> #########switch to GENCODE annotation, takes ~26s
> galn_custom_gencode <- Switchanno(galn_custom, gencode_v19)
> SummarizeproBAM(galn_custom_gencode)
> #########remove decoy PSMs
> proBAM_true_v <-  galn_custom_gencode[union(which(values(galn_custom_gencode)[['flag']] == 0),
+                                which(values(galn_custom_gencode)[['flag']] == 16))]
> #########Convert a PSMs based proBAM file to the peptide based proBAM file, takes ~1s
> pepBAM_true_v <- proBAM2pepBAM(proBAM_true_v)
> #########protein level parsimony, takes ~22min
> progp_res_v <- pepBAM_parsimony(proBAM_true_v, cdsByTx)
> #########gene level parsimony, takes ~6min
> genegp_res_v <- pepBAM_parsimony(proBAM_true_v, cdsByGe)
> #########keep protein group with >=2 peptides
> pg_v <- progp_res_v[which(as.integer(progp_res_v[, 4]) >=2), ]
> gegp_v <- genegp_res_v[which(as.integer(genegp_res_v[, 4]) >=2), ]
> dim(gegp_v)
> dim(pg_v)
> pg_names_v <- unlist(lapply(pg_v[, 1], function(x) strsplit(x, ':')[[1]][1]))
> gegp_names_v <- unlist(lapply(gegp_v[, 1], function(x) strsplit(x, ':')[[1]][1]))
> #########get count table for protein group, takes ~4s
> pcount_v <- proBAMCount(proBAM_true_v, cdsByTx)
> pcount_pg_v <- pcount_v[pg_names_v, ]
> #########get count table for gene group, takes ~3s
> gcount_v <- proBAMCount(proBAM_true_v, cdsByGe)
```

```
> gcount_gegp_v <- gcount_v[gegp_names_v, ]
> ##################################
> # COLO205 in TUM_NCI60 data set
> ##################################
> ## Size of proBAM file : 2.17MB
> bamFile <- '/TUM_NCI60/P001892.bam'
> #########takes ~4s
> galn_nci60 <- readproBAM(bamFile)
> #########takes ~10s
> SummarizeproBAM(galn_nci60)
> proBAM_true <- galn_nci60[union(which(values(galn_nci60)[['flag']] == 0),
+                              which(values(galn_nci60)[['flag']] == 16))]
> #########takes ~1s
> pepBAM_true <- proBAM2pepBAM(proBAM_true)
> #########takes ~43s
> progp_res_nci <- pepBAM_parsimony(pepBAM_true, cdsByTx)
> #########takes ~12s
> genegp_res_nci <- pepBAM_parsimony(pepBAM_true, cdsByGe)
> pg <- progp_res[which(as.integer(progp_res[, 4]) >=2), ]
> gegp <- genegp_res[which(as.integer(genegp_res[, 4]) >=2), ]
> dim(gegp)
> dim(pg)
> table(as.integer(pg[, 3]))
> table(as.integer(gegp[, 3]))
> pg_names <- unlist(lapply(pg[, 1], function(x) strsplit(x, ':')[[1]][1]))
> gegp_names <- unlist(lapply(gegp[, 1], function(x) strsplit(x, ':')[[1]][1]))
> #########takes ~5s
> pcount <- proBAMCount(proBAM_true, cdsByTx)
> #########takes ~4s
> gcount <- proBAMCount(proBAM_true, cdsByGe)
> pcount_pg <- pcount[pg_names, ]
> gcount_gegp <- gcount[gegp_names, ]
> ##################################
> # Merged data set
> ##################################
> #########combine data sets
> colon_combine <- c(proBAM_true_v, proBAM_true)
> #########takes ~20s
> SummarizeproBAM(colon_combine)
> proBAM_true_c <- colon_combine
> #########takes ~2s
> pepBAM_true_c <- proBAM2pepBAM(proBAM_true_c)
> #########takes ~ 110min
> progp_res_c <- pepBAM_parsimony(pepBAM_true_c, cdsByTx)
> #########takes ~20 min
```

```
> genegp_res_c <- pepBAM_parsimony(pepBAM_true_c, cdsByGe)
> pg_c <- progp_res_c[which(as.integer(progp_res_c[, 4]) >=2), ]
> gegp_c <- genegp_res_c[which(as.integer(genegp_res_c[, 4]) >=2), ]
> pg_names_c <- unlist(lapply(pg_c[, 1], function(x) strsplit(x, ':')[[1]][1]))
> gegp_names_c <- unlist(lapply(gegp_c[, 1], function(x) strsplit(x, ':')[[1]][1]))
> #########takes ~7s
> pcount_c <- proBAMCount(proBAM_true_c, cdsByTx)
> #########takes ~5s
> gcount <- proBAMCount(proBAM_true_c, cdsByGe)
> pcount_pg_c <- pcount_c[pg_names_c, ]
> gcount_gegp_c <- gcount[gegp_names_c, ]
>
>
```
